# Randomized, Double-Blind, Crossover Trial of Amitriptyline for Analgesia in Painful HIV-Associated Sensory Neuropathy

**DOI:** 10.1371/journal.pone.0126297

**Published:** 2015-05-14

**Authors:** Natalya Dinat, Edmore Marinda, Shirra Moch, Andrew S. C. Rice, Peter R. Kamerman

**Affiliations:** 1 Centre for Palliative Care, Faculty of Health Sciences, University of the Witwatersrand, Johannesburg, South Africa; 2 School of Public Health, Faculty of Health Sciences, University of the Witwatersrand, Johannesburg, South Africa; 3 Division of Pharmacology, School of Therapeutic Sciences, Faculty of Health Sciences, University of the Witwatersrand, Johannesburg, South Africa; 4 Pain Research, Department of Surgery and Cancer, Imperial College London, London, United Kingdom; 5 Pain Medicine, Chelsea and Westminster Hospital NHS Foundation Trust, London, United Kingdom; 6 Brain Function Research Group, School of Physiology, Faculty of Health Sciences, University of the Witwatersrand, Johannesburg, South Africa; University of Washington, UNITED STATES

## Abstract

We conducted a randomized, double-blind, placebo-controlled, crossover study at a single center in South Africa, to ascertain whether amitriptyline is an effective analgesic for painful HIV-associated sensory neuropathy of moderate to severe intensity in: i) antiretroviral drug naive individuals, and ii) antiretroviral drug users. 124 HIV-infected participants (antiretroviral drug naive = 62, antiretroviral drug users = 62) who met the study criteria for painful HIV-associated sensory neuropathy were randomized to once-daily oral amitriptyline (titrated to a median: interquartile range of 50: 25-50 mg) or placebo for six weeks, followed by a three-week washout period and subsequent treatment crossover. The primary outcome measure was change from baseline in worst pain intensity of the feet (measured by participant self-report using an 11-point numerical pain rating scale) after six weeks of treatment. 122 of 124 participants completed all study visits and were included in the analysis of the primary outcome. In the antiretroviral drug-naive group (n = 61) there was no significant difference in the mean change in pain score from baseline after six weeks of treatment with placebo or amitriptyline [amitriptyline: 2.8 (SD 3.3) vs. placebo: 2.8 (3.4)]. Similarly, there was no significant difference in the change in pain score after six weeks of treatment with placebo or amitriptyline in the antiretroviral drug-user group (n = 61) [amitriptyline: 2.7 (3.3) vs. placebo: 2.1 (2.8)]. Controlling for period effects and treatment order effects did not alter the outcome of the analyses. Nor did analyzing the intention-to-treat cohort (missing data interpolated using baseline observation carried forward) alter the outcome of the analyses. In summary, amitriptyline, at the doses used here, was no more effective than an inactive placebo at reducing pain intensity in individuals with painful HIV-associated sensory neuropathy of moderate to severe intensity, irrespective of whether they were on antiretroviral therapy or not.

**Trial Registration:**

ISRCTN 54452526

## Introduction

Symptomatic HIV-associated sensory neuropathy (HIV-SN) is a frequent complication of HIV infection and its treatment. Recent data from sub-Saharan Africa, the region worst affected by HIV [1,2], indicate that symptomatic neuropathy affects between 35 and 60% of ambulatory HIV-positive patients [3,4]. Moreover, the burden of HIV-SN is expected to remain high for the foreseeable future despite changes in the management of HIV [5–7]. The most salient symptom of the neuropathy is pain, which frequently is moderate to severe in intensity [3,7–9], and has been associated with reduced activities of daily living and physical function, sleep disruption, increased severity of depression and anxiety symptoms, and increased risk of being unemployed [7,9]. Yet, evidence for managing painful HIV-SN is poor [10–12].

Amitriptyline, is a tricyclic antidepressant that is effective in treating neuropathic pain [13], and is widely available in developing countries [2,14]. However, two published trials found amitriptyline was no more effective than placebo for the treatment of painful HIV-SN [15,16]. Both trials were stopped on the advice of their respective data monitoring boards because of lack of efficacy before recruitment targets were met. The trial by Shlay and co-workers [15] had poor blinding, and used a 2x2 factorial design with acupuncture and amitriptyline as interventions, which complicated the interpretation of the data. But the second study by Kieburtz and colleagues [16] was a high-quality parallel group study, with three interventions: placebo, amitriptyline and mexiletine. Despite these negative results, amitriptyline is recommended by international and national agencies for the treatment of painful HIV-SN [17,18], and is the only recommended first-line treatment for painful peripheral polyneuropathy included on the WHO essential medicines lists [2,13,19].

Because of this persistent recommendation of amitriptyline for the management of painful HIV-SN, we conducted a randomized-controlled trial to ascertain whether amitriptyline is a clinically effective analgesic for moderate to severe foot pain in individuals with HIV-SN. This trial differs from the previous two studies in that amitriptyline and placebo were the only interventions studied, simplifying the interpretation of results. Importantly, it included the separate investigation of the efficacy of the treatment in two patients groups; one group on stable antiretroviral therapy, and the other never exposed to antiretroviral therapy (both previous trials included mixed cohorts). This is critical, as there are no pathognomonic features to distinguish the polyneuropathy that develops in HIV-positive individuals before or after starting antiretroviral therapy despite likely differences in the mechanisms underlying the neuropathies [20]. The neuropathy that develops in patients never exposed to antiretroviral therapy is likely to be predominantly immune-mediated, while the neuropathy that develops soon after initiating antiretroviral therapy includes an additional insult by neurotoxic antiretroviral drugs such as stavudine (a drug still in common use in developing countries) [20,21]. It is unclear whether these mechanistic differences may alter responsiveness to therapy, but differences in the analgesic response to lamotrigine have been reported between patients with painful HIV-SN exposed to non-neurotoxic and neurotoxic antiretroviral therapy [22]. It is important to understand whether neuropathic pain caused by *either* mechanism may be amitriptyline responsive, with both likely contributing in many cases of painful, HIV-SN. Previous studies were underpowered for this purpose.

Thus, we have conducted a double-blind crossover trial of amitriptyline for analgesia in painful HIV-SN: i) in individuals never exposed to antiretroviral therapy, and ii) in individuals on stable antiretroviral therapy. We report that pain relief achieved by amitriptyline therapy did not differ from that achieved by placebo, irrespective of antiretroviral therapy exposure.

## Materials and Methods

The protocol for this trial and supporting CONSORT checklist are available as supporting information; see S1 CONSORT Checklist and S1 Protocol.

### Trial Design

We conducted a randomized, double-blind, placebo-controlled, crossover study comparing the analgesic efficacy of amitriptyline tablets to inactive placebo tablets, with a three-week washout period between interventions.

### Ethics statement

Permission to conduct the study was obtained from the Human Ethics Research Committee, University of the Witwatersrand (clearance number: M080709). Written informed consent was obtained from all participants. The trial was registered at the South African National Clinical Trials Register (NHREC#1188, issue date 4^th^ July 2008; registry is no longer operational), and International Standard Randomized Controlled Trial Number Register (ISRCTN54452526, issue date 17 November 2014).

### Participants

The study took place at a single site, Chris Hani Baragwanath Hospital, Soweto, South Africa, between 23 April 2009 and 17 November 2009. Eligible participants were aged 18 years or older, had a confirmed HIV infection, and met the criteria for current symptomatic HIV-SN according to the validated Brief Peripheral Neuropathy Screening Tool [23]. The tool requires the bilateral presence of at least 1 symptom (pain, aching, burning, numbness, or pins-and-needles) and at least 1 clinical sign of neuropathy (reduced vibration sense or absent ankle reflexes) for a diagnosis of symptomatic HIV-SN to be made. Vibration sense was assessed using a 128 Hz tuning fork, which was placed on the interphalangeal joint of each great toe; perception of vibration sense for 10 seconds or less was considered abnormal. Trial participants were required to have moderate to severe pain (score ≥4 on an 11-point numerical pain rating scale) in both feet over the previous three days. [24].

Participants had to be either on stable antiretroviral therapy for more than 6 months (ARV-user group), or antiretroviral therapy naïve (ARV-naïve group). Exclusion criteria were: severe pain from HIV-SN that warranted a change in treatment regimen; already taking amitriptyline or having taken the drug in the previous three weeks; limb amputation; Kaposi sarcoma of the lower limbs; current post-herpetic neuralgia or herpes zoster; pregnancy or the intention of falling pregnant; receiving treatment for tuberculosis; malignancy not related to HIV; major psychiatric disorders; epilepsy; use of monoamine oxidase inhibitors, other antidepressants or anti-epileptic drugs; renal failure requiring intervention; diabetic neuropathy; clinically significant liver failure or a history of liver failure; extreme pain or exhaustion; recent myocardial infarction, arrhythmias or heart block; a history of urinary retention, urinary hesitancy or closed angle glaucoma; and participation in another trial or study.

### Intervention

Participants attended six scheduled visits every three weeks over 15 weeks. This comprised six weeks on intervention A (amitriptyline) or B (placebo), three weeks washout and then crossover to six weeks intervention on B or A respectively. Participants took the assigned medication daily during each six-week intervention. At the baseline visit, participants were provided with a three-week supply of medication. Over a maximum of two weeks, dose escalation occurred to tolerance (participants were not able to tolerate perceived side-effects) or effect (participants achieved significant pain relief) every three days, based on telephonic conversation between the participant and a study nurse. The dose escalation schedule was as follows: 25mg, 50mg, 75mg, 100mg, 150mg (maximum dose) formulated as single 25mg tablets for amitriptyline or one, two, three, four or six tablets of placebo. The maximum tolerated dose achieved was taken by each participant for the remainder of the six-week period.

At the end of week three, the participants visited the study center, returned unused drugs and were issued with a second container of tablets containing the correct maximum daily dose for each participant for the remaining three weeks of the initial treatment phase. At the clinic visit at the end of week six, participants returned all unused drugs, and were questioned on the number of missed doses. They were also advised on the three-week washout period. The duration of the washout period was calculated based on the pharmacokinetics of amitriptyline [25]. The use of pre-specified rescue medication (acetaminophen, non-steroidal anti-inflammatory drugs, codeine phosphate) was permitted. After the washout period, the above procedures were repeated, but the interventions were switched.

### Outcomes

The primary outcome measure was the difference in pain intensity of the feet at baseline and at six weeks of intervention, as measured by participant self-report using an 11-point numerical pain rating scale [26]. Specifically, pain intensity referred to the pain in the feet at the time of each of the six study visits (the convention of asking “average pain” has been shown to perform poorly in this population [27]). Dose escalation and maximum dosage of amitriptyline used was noted, as were side effects and adverse events. The use of rescue medication, including prescribed, over-the-counter and self-administered treatments, as well as traditional medicine and treatments, was recorded. No participants were taking gabapentin, pregabalin, carbamazepine or lamotrigine at enrolment or used these agents during the course of the study as rescue medications.

### Sample Size

To detect a clinically meaningful reduction in the intensity of peripheral neuropathy of 2 units on an 11-point NRS [10,13,24], assuming 90% power, 5% level of significance and assuming common variance in the two crossover periods of 2, we estimated that 56 individuals would be required. We assumed a loss to follow-up of 10%, thus increased the sample size to 62 participants in each of the ARV-naive and ARV-user groups.

### Randomization and Masking

The random allocation sequence was determined using a randomized block model. For allocation of the treatment sequence, a computer-generated list of alphanumeric identity numbers was used for all 124-treatment packs. EM generated the random allocation sequence, and assignment of interventions was by SM. The active and placebo tablets were identical in appearance. The study doctor, study nurse (responsible for enrolment) and participants were blinded to the intervention. The drug used was Trepiline (Aspen Pharmacare, Port Elizabeth, South Africa). An identical-looking placebo was manufactured by Azochem Laboratories (Roodepoort, South Africa). Placebo tablets contained microcrystalline cellulose and sorbitol.

### Statistical Methods

Primary analysis was performed on a per protocol basis. Analyses were conducted separately for each of the two participant groups (ARV-naive and ARV-user), and for all participants combined. Pain intensity scores are reported as mean (SD). Crude statistical analyses of pain scores at the start of each intervention (baseline scores) were carried out using paired and unpaired t-tests, with Bonferroni correction for multiple comparisons. Analysis of the primary outcome included crude comparisons of change in pain score over six weeks of treatment with amitriptyline or placebo using paired t-tests (ignoring period or treatment order effects), and using ANOVA to account for possible period effects and treatment order effects in the model. The ANOVA analyses were repeated using the intention-to-treat population, using the baseline observation carried forward method to interpolate missing values.

## Results

### Participant Flow

**Fig 1:**
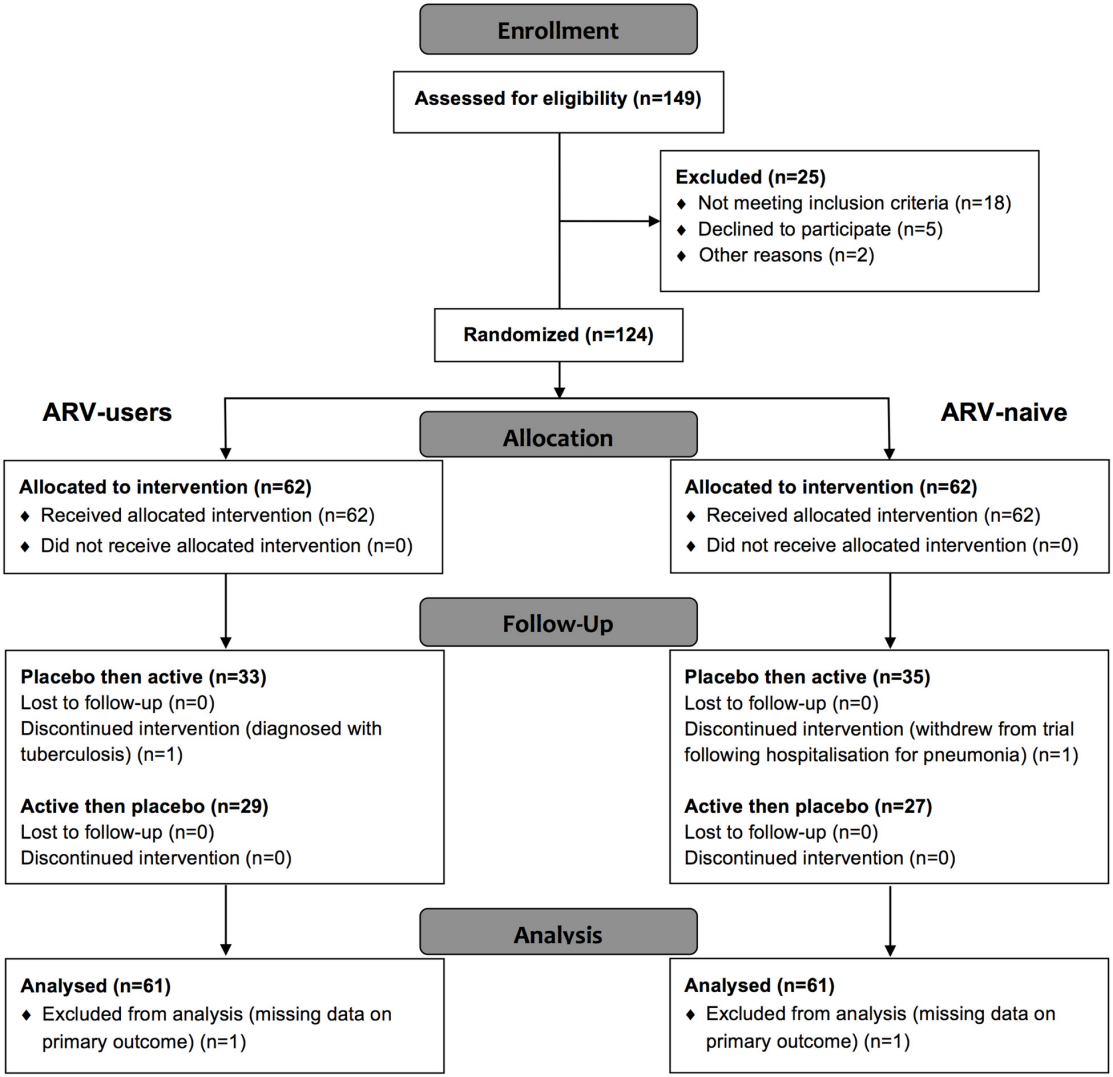
CONSORT diagram illustrating participant flow during the study. The two randomized participants whose data were excluded from the analysis dropped out of the trial for non-trial related reasons: one participant (ARV-user) was disenrolled from the study to start tuberculosis therapy after her test results, which had been misplaced and then found, showed that she had active pulmonary tuberculosis; and one participant (ARV-naive) was lost to follow-up after being hospitalized for the treatment of community acquired pneumonia.

Participant flow is summarized in Fig 1. One hundred and twenty-four (124) participants were randomized to treatment; 122 completed all 6 study visits and were included in the per protocol analyses. Two participants lost their tablets and missed three and two days of treatment, respectively, their data were included in the analyses. There were no missing data on the primary end-point for 122 participants included in the analyses.

### Demographic Data

Participant baseline demographic data are shown in Table 1. All participants were of black African descent, 71% were females, and the mean age was 38 years. Amongst the ARV-user group, 62% had been exposed to stavudine. Participants in the ARV-user group tended to be older, had fewer years of formal schooling, and had lower CD4 T-cell counts than participants in the ARV-naïve group.

**Table 1:** Baseline characteristics of participants (per protocol cohort, n = 122).

	Allparticipants (n = 122) [n (%)]	ARV-naïve (n = 61) [n (%)]	ARV-user (n = 61) [n (%)]
**Female**	87 (71)	42 (69)	45 (74)
**Age (years)** ^**1**^	38 (8.9)	34 (8.6)	42 (7.4)*
**≥ 9 years of education** ^**2**^	90 (77)	52 (88)	38 (66)*
**Employed (part-time, full-time or temporary)**	117 (96)	57 (93)	60 (98)
**CD4 T-cell count** ^**3**^ ^,^ ^**4**^	318 (203–461)	450 (331–614)	211 (125–305)*
**Stavudine use:**			
*Ever*	-	-	44 (72)
*Current*	-	-	38 (62)

^1^ Mean (standard deviation)

^2^ n = 117 for all participants, n = 59 for ARV-naïve and n = 58 for ARV-user

^3^ Median (IQR)

^4^ n = 116 for all participants, n = 57 for ARV-naïve and n = 59 for ARV-user

* Statistically different to ARV-naïve at 5% significance level

### Dosage Titration

In the ARV-user group there was no difference in titration dose between amitriptyline and placebo; participants were titrated to a median of 2 (IQR: 1–2) tablets of per day (drug dose median: 50mg, IQR: 25–50mg) when administered amitriptyline, and a median of 2 (IQR: 1–2) tablets per day when administered placebo (Wilcoxon signed-rank V = 386, p = 0.59). In the ARV-naïve group, there was no difference in median titration dose between amitriptyline and placebo; participants were titrated to a median of 2 (IQR: 1–2) tablets of per day (drug dose median: 50mg, IQR: 25–50mg) when administered amitriptyline, and a median of 2 (IQR: 1–3) tablets per day when administered placebo (Wilcoxon signed-rank V = 415, p = 0.84). There was no difference between the ARV-user and ARV-naïve groups in the median titration dose for amitriptyline (Wilcoxon rank sum W = 1643, p = 0.24) and placebo (Wilcoxon rank sum W = 1757, p = 0.58).

### Primary Outcome

**Fig 2:**
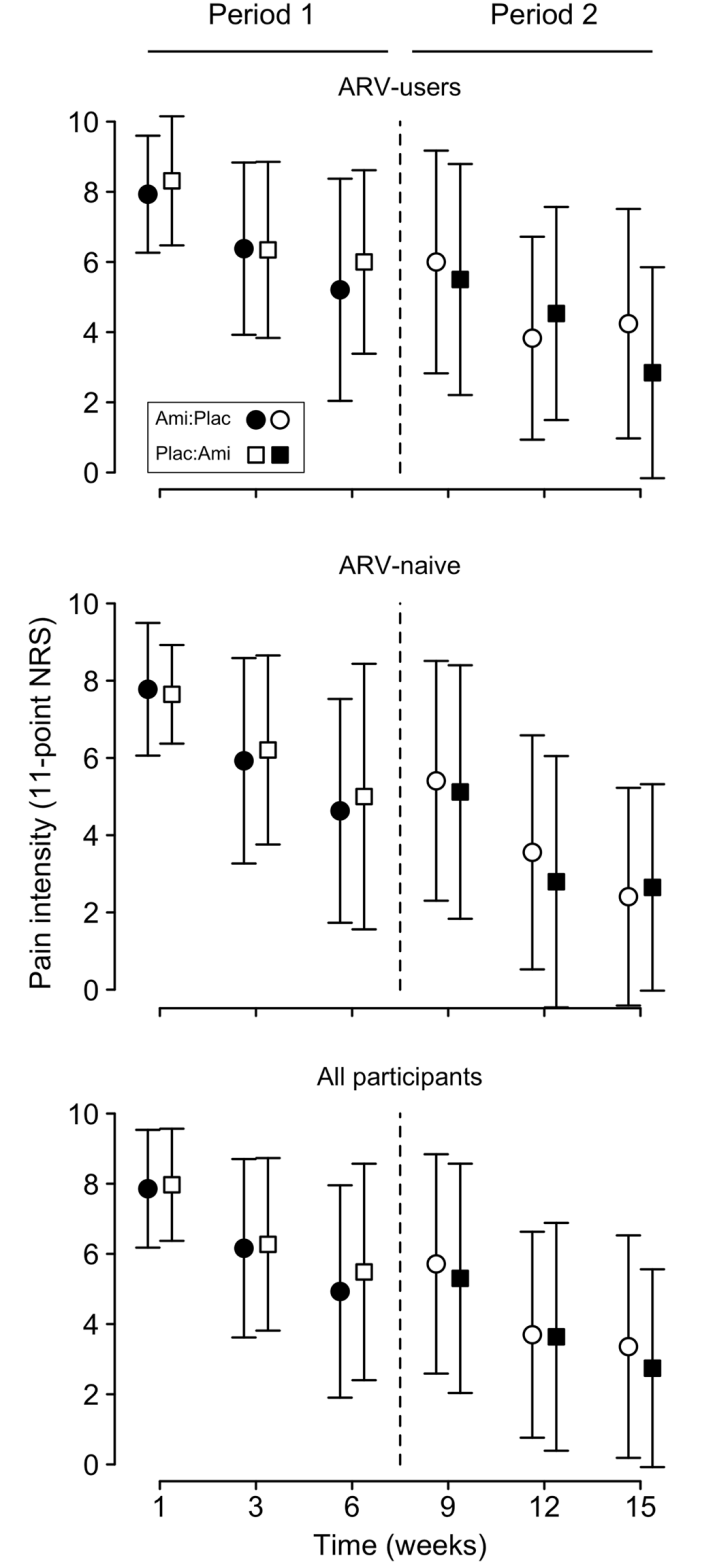
Mean (SD) pain intensity across the trial for ARV-users, ARV-naïve and all participants. The figure includes the two 6-week intervention periods (Period 1 and Period 2) for ARV-users (top panel), ARV-naïve (middle panel), and for all participants irrespective of ARV exposure (bottom panel). In all three panels, circles show data from participants who were randomized to receive amitriptyline in period 1 (● amitriptyline) and placebo in period 2 (○placebo), squares show data from participants who were randomized to receive placebo in period 1 (☐placebo) and amitriptyline in period 2 (■amitriptyline). There was no statistical difference in treatment efficacy between amitriptyline and placebo within each group (see text, Table 2, and S1 Table for details).

Fig 2 shows pain scores across the two periods of the trial for the ARV-user and ARV-naïve groups, and all participants combined. There were no significant differences in mean pain scores for ARV-users receiving amitriptyline or placebo at the start of week 1 (baseline for period 1) (amitriptyline: 7.9, SD 1.7; placebo: 8.3, SD 1.8; t_(59)_ = -0.85, p = 0.40), or at the start of week 9 (baseline for period 2) (amitriptyline: 5.5, SD 3.3; placebo: 6.0, SD 3.2; t_(59)_ = -0.60, p = 0.55). However when ignoring intervention, there was a significant period effect, such that pain scores at the start of week 9 were significantly less than at the start of week 1 (period 1: 8.1, SD 1.8; period 2: 5.7, SD 3.2; t_(60)_ = 5.26, p < 0.001). There were no significant differences in mean pain scores for ARV-naïve participants receiving amitriptyline or placebo at the start of week 1 (amitriptyline: 7.8, SD 1.7; placebo: 7.6, SD 1.3; t_(59)_ = 0.34, p = 0.73), or at the start of week 9 (amitriptyline: 5.1, SD 3.3; placebo: 5.4, SD 3.1; t_(59)_ = -0.35, p = 0.73). However, there was a significant period effect, such that pain scores at the start of week 9 were significantly less than at the start of week 1 (period 1: 7.7, SD 1.5; period 2: 5.2, SD 3.2; t_(60)_ = 5.60, p < 0.001). There were no significant differences in baseline pain scores between the ARV-user and ARV-naïve groups at week 1 (ARV-users: 8.1, SD 1.8; ARV-naive: 7.7, SD 1.5; t_(120)_ = 1.45, p = 0.15) or week 9 (ARV-users: 5.7, SD 3.2; ARV-naive: 5.2, SD 3.2; t_(120)_ = 0.85, p = 0.40).

Ignoring any potential period or treatment order effects, there was no significant difference in the absolute change in pain score over six weeks of treatment with placebo or amitriptyline in the ARV-user group (amitriptyline: 2.7, SD 3.3; placebo: 2.1, SD 2.8; t_(60)_ = -1.13, p = 0.26), the ARV-naïve group (amitriptyline: 2.8, SD 3.3; placebo: 2.8, SD 3.4; t_(60)_ = 0.05, p = 0.96), or all participants combined (amitriptyline: 2.7, SD 3.2; placebo: 2.4, SD 3.2; t_(121)_ = -0.72, p = 0.47). Summaries of the ANOVA analyses, which controlled for period and order effects, are shown in Table 2. For all analyses, no significant treatment effects (changes in pain scores did not differ significantly between amitriptyline and placebo treatment) or order effects (the sequence in which placebo and amitriptyline were taken did not influence changes in pain scores) were detected. But, significant period effects (pain scores in period 2 were lower than in period 1) and significant time effects (pain scores decreased over time within each period) were detected. Intention-to-treat analysis of the full cohort of 124 randomized participants, using baseline observation (from period 1) carried forward, did not alter the result (S1 Table).

**Table 2:** ANOVA summary (per protocol cohort, n = 122).

	df	F-value	p-value
**ARV-USER**			
**Between group:**			
*Order* ^*1*^	1, 59	0.01	0.99
**Within group:**			
*Period* ^*2*^	1, 301	93.8	< 0.001*
*Time* ^*3*^	2, 301	37.3	< 0.001*
*Treatment* ^*4*^	1, 301	2.7	0.09
**ARV-NAIVE**			
**Between group:**			
*Order* ^*1*^	1, 59	0.01	0.92
**Within group:**			
*Period* ^*2*^	1, 301	116.6	< 0.001*
*Time* ^*3*^	2, 301	47.6	< 0.001*
*Treatment* ^*4*^	1, 301	0.86	0.35
**ALL PARTICIPANTS**			
**Between group:**			
*Order* ^*1*^	1, 119	0.02	0.88
*ARV* ^*5*^	1, 119	3.8	0.05
**Within group:**			
*Period* ^*2*^	1, 606	210.8	< 0.001*
*Time* ^*3*^	2, 606	84.9	< 0.001*
*Treatment* ^*4*^	1, 606	3.5	0.06

^1^ Order of treatment

^2^ Period 1 vs. period 2

^3^ Weeks

^4^ Amitriptyline vs. placebo

^5^ ARV-user vs. ARV-naïve

* Statistically significance

To avoid the period effect, and because we found no meaningful differences between the ARV-user and ARV-naïve groups, we analyzed the data as a parallel two-arm study using the data from the first period only (weeks 1 to 6). When data from the ARV-user and ARV-naïve groups were combined we did not detect a treatment effect (amitriptyline vs placebo), but there was a significant decrease in pain intensity in both treatment groups over the six-week period, such that pain intensity decreased from 7.9 (SD 1.7) to 4.9 (SD 3.0) in the group receiving amitriptyline (n = 56), and from 8.0 (SD 1.6) to 5.5 (SD 3.1) in the placebo group (n = 66) (ANOVA results are summarized in S2 Table).

We also calculated the number needed to treat to achieve at least 50% pain relief using data from all participants in period 1 and data from period 2 for participants who started week 9 (baseline for period 2) with at least moderate pain (≥ 4 on the 11-point NRS). Forty-seven (47) of 105 participants achieved at least 50% pain reduction over the six-week treatment period when taking amitriptyline, while 42 of 110 participants achieved at least 50% pain relief when taking placebo (NNT: 16, 95% CI: 5.1 to -15.2). Because the 95% CI of the NNT includes negative values, the NNT may be interpreted as: amitriptyline was helpful (compared to placebo), and the number needed to treat is greater than 5.1, or amitriptyline treatment was harmful (compared to placebo), and the number needed to harm is greater than 15.2.

We observed no dose-response relationship between the dose of amitriptyline taken and the magnitude of the change in pain intensity over six weeks of treatment (S1 Data).

### Safety and Adverse Events

Adverse events are reported in S3 Table. Treatment was well tolerated at the dosages administered. The three most common adverse events observed during the study were drowsiness, dry mouth and chest pain, which were common to the use of amitriptyline and placebo. Significantly more participants on the amitriptyline arm reported dry mouth compared to when taking placebo.

### Rescue Medication

The use of rescue medications in the last week of each six-week treatment period was analyzed. Data from the ARV-user and ARV-naïve groups were combined for these analyses. In brief, 13 participants (11%) in each of the placebo and amitriptyline arms of the trial indicated that had taken rescue medication for their pain in the last week of each intervention (McNemar’s p-value = 1). Despite the low proportion of patients indicating that they had taken rescue medication for pain, the majority of participants’ complete lists of medications they were taking (for any reason), included analgesics and anti-inflammatory agents (data summarized in S4 Table).

## Discussion

We conducted a randomized, placebo-controlled trial to evaluate the efficacy of amitriptyline for the treatment of moderate to severe pain in participants with HIV-SN. In participants on stable antiretroviral therapy (ARV-user group) and in those who had never been exposed to antiretroviral therapy (ARV-naïve group), there was no significant difference in the magnitude of the pain relief produced by amitriptyline compared to placebo after six weeks of treatment. Treatment order did not influence the primary outcome. A period effect was seen for amitriptyline and placebo treatment following the washout period, with pain intensities not returning to baseline values after treatment withdrawal.

Our study is novel in its investigation of the efficacy of amitriptyline in two patient groups: i) an ARV-naïve group, whose neuropathy is presumed to me immune-mediated, and ii) an ARV-user group, whose neuropathy may include an additional iatrogenic insult to peripheral nerves from the antiretroviral drugs. These two groups reflect the two broad classes of patients attending outpatient HIV care clinics, and despite possible differences in the mechanisms underlying the polyneuropathy they develop (immune versus immune and drug toxicity) no studies on analgesic therapies for painful HIV-SN have differentiated between these two groups. Also, we conducted the study in a cohort of African descent, set in a socio-economic environment that is broadly reflective of those encountered by most HIV-infected individuals in Southern Africa; the region worst affected by HIV. Our finding that amitriptyline is no better than placebo in ARV-users and ARV-naïve patients is significant for the clinical management of painful HIV-SN, especially in developing countries where the burden of HIV and therefore HIV-SN is high. Agencies providing clinical guidance need to decide whether amitriptyline, should continue to be recommended in the treatment of HIV-SN.

Our findings support those of two previous trials, both of which did not achieve the calculated sample size after being stopped on the advice of their respective data monitoring boards because of lack of efficacy before recruitment targets were met [15,16]. However, the potential clinical effectiveness of amitriptyline was difficult to assess in the those two trials because enrolment included participants with mild pain, and the Initiative on Methods, Measurement, and Pain Assessment in Clinical Trials (IMMPACT) [24] recommend that pain of moderate to severe intensity be investigated. Furthermore, in the study conducted by Shlay and colleagues [15] the investigators had to deviate from the original study design because of issues related to randomization, and their 2x2 factorial design with acupuncture and amitriptyline interventions complicated interpretation. Thus our trial is the first completed study to investigate the efficacy of amitriptyline in moderately to severely painful HIV-SN. Moreover, it is also the first trial of amitriptyline to show that the agent is not superior to placebo in the painful neuropathy that develops in participants who have not been exposed to antiretroviral therapy.

While the average results in the ARV-user and ARV-naïve groups were no additional analgesic effect for amitriptyline above that achieved by placebo, 42% (26/61) of participants in both groups achieved pain relief of at least 2 points (on the 11-point numerical pain rating scale) greater than that achieved when they were taking placebo. Analysis of disease, demographic, and phenotype characteristics that may differentiate these responders from non-responders in each group, did not yield any significant findings, except in the ARV-naïve group, where responders tended to be younger than non-responders (S1 and S2 Data). A study of the analgesic effects of pregabalin in painful HIV-SN reported that responders were more likely to have increased sensitivity to a pin-prick stimulus compared to non-responders [28], but unfortunately we only assessed for pin-prick hypoesthesia and not hyperesthesia.

The convention in analgesic trials is to measure “average pain”, but we measured “current pain” at the time of each interview as our primary outcome. Our reason for using “current pain” is the poor performance of “average pain” in this population [27]. While “current pain” is susceptible to circadian changes [29,30], participants in this study were seen in a six-hour window between 09:00 and 15:00, thus limiting the effect any diurnal variation in pain intensity may have had. Also, reanalysis of the data using “average pain over the past three days” did not change the interpretation of the data (S3 and S4 Data).

We used a crossover design for this study. Whilst period and treatment-order effects have not been noted in other neuropathy studies employing a crossover design [31] we found that the reduction in pain scores continued to be observed between treatment periods, making interpretation of the results difficult. From a pharmacokinetics perspective the three-week wash out period was adequate. Moreover, when we combined the participants in the ARV-user group and ARV-naive groups (exposure status to antiretroviral therapy did not influence treatment effect, Table 2) and looked only at the first six-week period to obviate the period effect, we still found that amitriptyline was no more effective than placebo in reducing HIV-SN pain intensity. Nevertheless, parallel-group studies may be a better option for future studies to avoid this complication in pain studies in particular.

Our trial found that inactive placebo produced similar pain relief to that of amitriptyline. Emerging research reports that the placebo effect is particularly prevalent in chronic pain and appears to be high in HIV-positive participants; in the current study, 38% of participants with moderate pain achieved at least 50% pain relief when on placebo, which is similar to the placebo responder rate of 42% recently reported in a trial of pregabalin in painful HIV-SN [28]. A recent meta-analysis of placebo responder rates in drug trials of painful neuropathy reported that trials on patients with painful HIV-SN show significantly greater placebo responder rates than other causes of neuropathic pain [32]. Indeed, the strong period effect in pain relief we observed may well indicate a persistent placebo response in our group of participants. This sustained pain relief may have been triggered through non-conscious cues, for example continued interaction with investigators [33]. This therapeutic “care effect” has been reported in a variety of clinical situations [34–36].

To mimic clinical practice, we used a flexible dose study design with dose escalation starting at 25mg per day, and a median dose of 50mg per a day was achieved in both study groups. Although the median dose of amitriptyline achieved was within the dose range recommended by Attal and colleagues [19], meta-analyses of studies employing amitriptyline for the treatment of neuropathic pain report that the average dose of amitriptyline being taken in trials where amitriptyline was deemed to be superior to placebo was 90 mg per day (range: 65–150 mg per day) [37–39]. Thus, participants in the current study may have been taking too low a dose of amitriptyline to achieve pain relief superior to placebo.

We escalated the dose of amitriptyline to tolerance or effect, and certainly, there was a significant decrease in pain intensity over each six-week intervention period. This decrease in pain intensity over time, irrespective of treatment, may have led to suboptimal dose escalation for amitriptyline. Indeed, it is unlikely that side effects were a significant cause of participants failing to escalate the dose of amitriptyline being taken. Moore and colleagues [39] recently reported that 64% of participants taking amitriptyline had at least one side effect (compared to 40% taking placebo), a rate that is far greater than the very low rate of side effects we detected in our study cohort. The most plausible explanation for the low side effect rate in this study cohort is that participants were taking too low a dose of amitriptyline. Alternatively, the low side effect rate in this study may have been caused by malabsorption of the drug or non-adherence to the treatment. We did not measure plasma levels of amitriptyline, so we cannot exclude either possibility, but only two participants were noted to have missed doses when pill counts were conducted at weeks three and six for each intervention period. A possible confounder when assessing tolerability of amitriptyline in HIV-positive individuals is the occurrence of dry mouth and fatigue in up to 72% and 48% of ambulatory HIV-infected individuals, respectively [40]. Thus, study participants may not regard these symptoms as new, leading to under-reporting of side effects.

## Conclusions

We have shown in this randomized controlled study that amitriptyline, which is widely used and recommended for this indication, is no more effective than inactive placebo for the treatment of moderate to severe HIV-SN pain, irrespective of whether participants were on antiretroviral therapy or not. While we cannot exclude the possibility that a significant treatment effect may have been observed at higher doses of amitriptyline, we believe that our findings are robust, and that the doses attained during drug titration reflect those used in clinical practice in our setting (unpublished data).

We believe that our and others’ failure to show efficacy for amitriptyline in a variety of different settings is of significant concern given that amitriptyline is recommended by several agencies [17,18] for the treatment of painful HIV-SN, and is the only drug recommended first line for the treatment of neuropathic pain that is included on the WHO master list of essential medicines and the essential medicines lists of most developing countries [2,14]. Further research is urgently required to understand the mechanisms and pathophysiology of HIV-SN and to develop alternative treatment modalities and evaluate preventative strategies.

## Supporting Information

S1 CONSORT Checklist CONSORT checklist. (PDF)

S1 Data Frequency distribution of amitriptyline tablets taken per day, and the relationship between dose of amitriptyline and pain relief (per protocol cohort: n = 122). (PDF)

S2 Data Characteristics of responders in ARV users (per protocol cohort: n = 61). (PDF)

S3 Data Characteristics of responders in ARV-naïve participants (per protocol cohort: n = 61). (PDF)

S4 Data ANOVA summary and data plot for “average pain in the last 3 days” in ARV-users (intention-to-treat cohort: n = 62). (PDF)

S5 Data ANOVA summary and data plot for “average pain in the last 3 days” in ARV-naïve participants (intention-to-treat cohort: n = 62). (PDF)

S1 Protocol Trial protocol. (PDF)

S1 Table ANOVA summary (intention-to-treat cohort, n = 124). (PDF)

S2 Table ANOVA summary for period 1 comparison of amitriptyline and placebo-treated participants (per protocol cohort, n = 122). (PDF)

S3 Table Adverse events during amitriptyline and placebo treatment (n = 122). (PDF)

S4 Table Rescue medications (per protocol cohort, n = 122). (PDF)
