# Supplementary material for: Randomized, Double-Blind, Crossover Trial of Amitriptyline for Analgesia in Painful HIV-Associated Sensory Neuropathy
Source: PLoS One. 2015 May 14;10(5):e0126297. doi: 10.1371/journal.pone.0126297 (PMC4431817; doi:10.1371/journal.pone.0126297)

S1 Data. Frequency distribution of amitriptyline tablets taken per day, and the relationship between dose of amitriptyline and pain relief (per protocol cohort: n = 122)

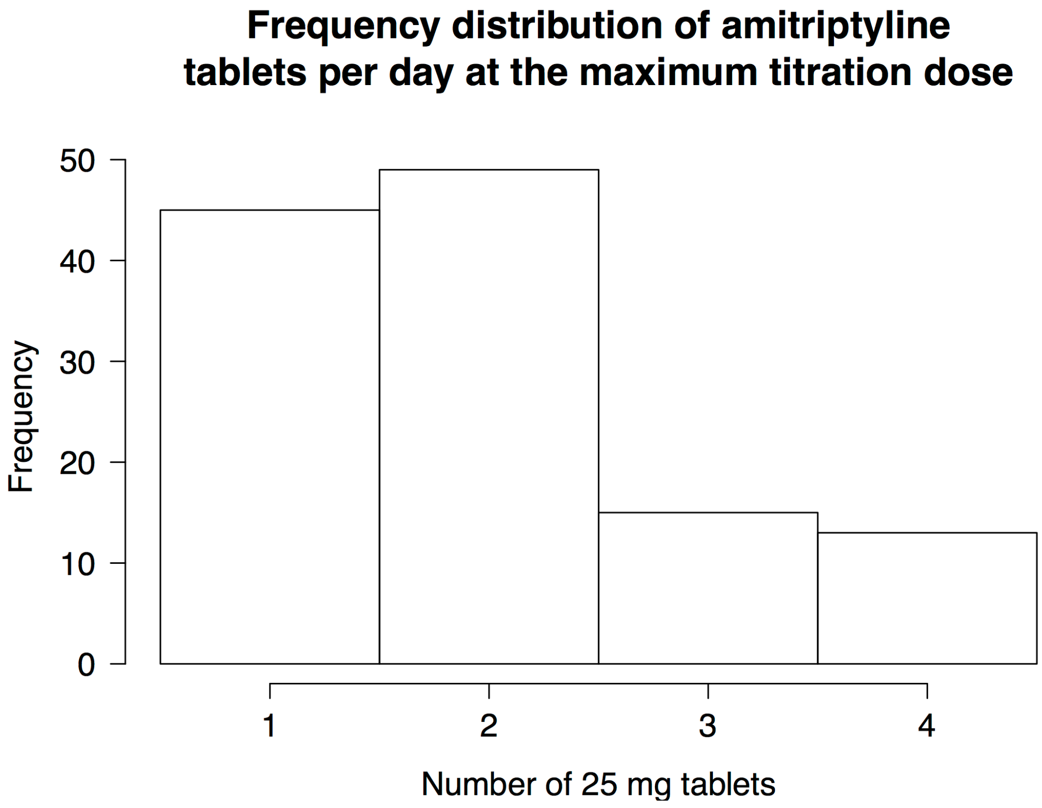

Plots of maximum titration dose of amitriptyline versus change in pain intensity over six weeks of treatment

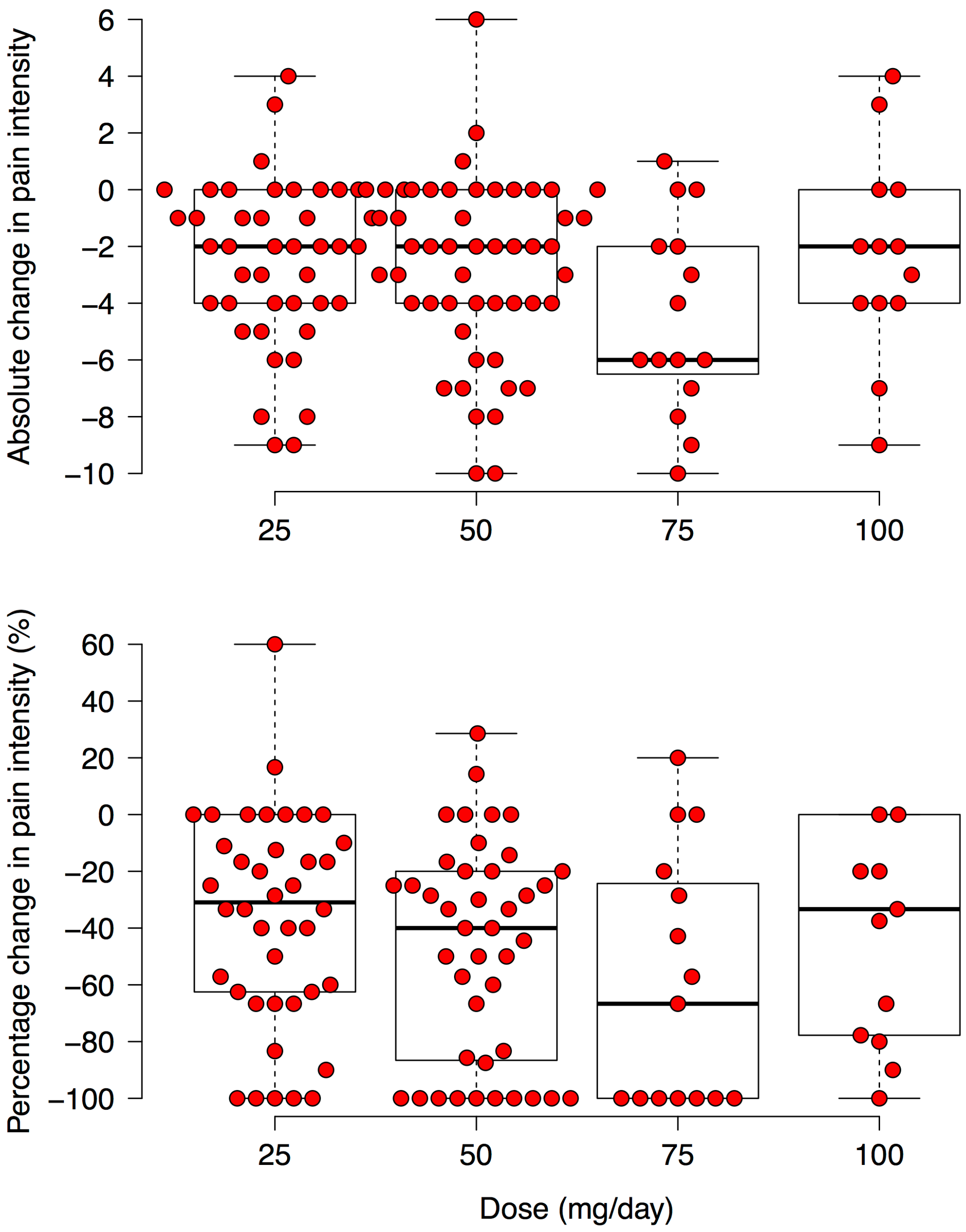

Supplement: S1 Data — (PDF) [file pone.0126297.s002.pdf]
