# Supplementary material for: Randomized, Double-Blind, Crossover Trial of Amitriptyline for Analgesia in Painful HIV-Associated Sensory Neuropathy
Source: PLoS One. 2015 May 14;10(5):e0126297. doi: 10.1371/journal.pone.0126297 (PMC4431817; doi:10.1371/journal.pone.0126297)
Supplement: S2 Data — (PDF) [file pone.0126297.s003.pdf]

S2 Data. Characteristics of responders in ARV users (per protocol cohort: n = 61)

Responders were defined as those participants with a change in pain score (between baseline and week six of intervention) of two or more points on the 11-point NRS when administered amitriptyline compared to when they were admnistered placebo.

Number of responders = 26 (42%)

Dose of amitriptyline

	Median (IQR) dose in mg
Non-responder	50 (25 - 50)
Responder	50 (25 - 75)

Wilcoxon rank sum test: Dose by Responder
W = 369, p-value = 0.18

Order of interventions
(AP: Amitriptyline then Placebo, PA: Placebo then Amitriptyline)

	Order	
	AP	PA
Non-responder	17	18
Responder	12	14

Fisher's Exact Test: Responder and Order
p-value = 1; Odds ratio (95% CI) = 1.1 (0.35 to 3.44)

Age

	Mean (SD) age in years
Non-responder	41.0 (7.51)
Responder	42.9 (7.28)

Welch Two Sample t-test: Age by Responder
t = -1.00, df = 53, p-value = 0.32

Sex

	Female	
	No	Yes
Non-responder	9	26
Responder	7	19

Fisher's Exact Test: Responder and Female
p-value = 1; Odds ratio (95% CI) = 0.9 (0.26 to 3.55)

CD4 T-cell count

	Median (IQR) CD4 T-cell count
Non-responder	195 (125 to 260)
Responder	247 (123 to 321)

Wilcoxon rank sum test: CD4 by Responder
W = 371; p-value = 0.41

Ever used stavudine
(D4T: stavudine)

	D4T ever	
	No	Yes
Non-responder	11	24
Responder	6	20

Fisher's Exact Test: Responder and D4T ever
p-value = 0.596; Odds ratio (95% CI) = 1.5 (0.42 to 5.94)

Employment

	Employed	
	No	Yes
Non-responder	1	34
Responder	0	26

Fisher's Exact Test: Responder and Employed
p-value = 1; Odds ratio (95%CI) = inf (0.02 to inf)

Education
(≥ 9 years of education)

	Education ≥ 9 years	
	No	Yes
Non-responder	8	24
Responder	12	14

Fisher's Exact Test: Responder and Education ≥ 9 years
p-value = 0.106; Odds ratio (95%CI) = 0.4 (0.11 to 1.35)

Symptoms: burning

	Burning	
	No	Yes
Non-responder	6	29
Responder	6	20

Fisher's Exact Test: Responder and Burning
p-value = 0.75; Odds ratio (95% CI) = 0.7 (0.16 to 3.01)

Symptoms: painful cold

	Painful cold	
	No	Yes
Non-responder	2	33
Responder	3	23

Fisher's Exact Test: Responder and Painful cold
p-value = 0.64; Odds ratio (95% CI) = 0.47 (0.04 to 4.45)

Symptoms: electric shocks

	Electric shocks	
	No	Yes
Non-responder	11	24
Responder	4	22

Fisher's Exact Test: Responder and Electric shocks
p-value = 0.23; Odds ratio (95% CI) = 2.48 (0.62 to 12.30)

Symptoms: tingling

	Tingling	
	No	Yes
Non-responder	4	31
Responder	1	25

Fisher's Exact Test: Responder and Tingling
p-value = 0.38; Odds ratio (95% CI) = 3.2 (0.29 to 164.96)

Symptoms: pins and needles

	Pins and needles	
	No	Yes
Non-responder	2	33
Responder	1	25

Fisher's Exact Test: Responder and Pins and needles
p-value = 1; Odds ratio (95% CI) = 1.5 (0.07 to 92.86)

Symptoms: numbness

	Numbness	
	No	Yes
Non-responder	2	33
Responder	1	25

Fisher's Exact Test: Responder and Numbness
p-value = 1; Odds ratio (95% CI) = 1.5 (0.07 to 92.86)

Symptoms: itching

	Itching	
	No	Yes
Non-responder	6	29
Responder	10	16

Fisher's Exact Test: Responder and Itching
p-value = 0.1; Odds ratio (95% CI) = 0.3 (0.08 to 1.25)

Signs: touch hypoaesthesia

	Touch hypoaesthesia	
	No	Yes
Non-responder	33	2
Responder	24	2

Fisher's Exact Test: Responder and Touch hypoaesthesia
p-value = 1; Odds ratio (95% CI) = 1.4 (0.09 to 20.11)

Signs: pin-prick hypoaesthesia

	Pin.prick.hypoesthesia	
	No	Yes
Non-responder	11	24
Responder	15	11

Fisher's Exact Test: Responder and Pin-prick hypoaesthesia
p-value = 0.066; Odds ratio (95% CI) = 0.3 (0.10 to 1.09)

Signs: brush allodynia

	Brush allodynia	
	No	Yes
Non-responder	25	10
Responder	20	6

Fisher's Exact Test: Responder and Brush.allodynia
p-value = 0.77; Odds ratio (95% CI) = 0.8 (0.19 to 2.77)
