# Supplementary material for: Randomized, Double-Blind, Crossover Trial of Amitriptyline for Analgesia in Painful HIV-Associated Sensory Neuropathy
Source: PLoS One. 2015 May 14;10(5):e0126297. doi: 10.1371/journal.pone.0126297 (PMC4431817; doi:10.1371/journal.pone.0126297)
Supplement: S3 Data — (PDF) [file pone.0126297.s004.pdf]

S3 Data. Characteristics of responders in ARV-naïve participants (per protocol cohort: n = 61)

Responders were defined as those participants with a change in pain score (between baseline and week six of intervention) of two or more points on the 11-point NRS when administered amitriptyline compared to when they were admnistered placebo.

Number of responders = 26 (42%)

Dose of amitriptyline

	Median (IQR) dose in mg
Non-responder	50 (25 - 50)
Responder	50 (31.25 – 68.75)

Wilcoxon rank sum test: Dose by Responder
W = 425, p-value = 0.55

Order of interventions
(AP: Amitriptyline then Placebo, PA: Placebo then Amitriptyline)

	Order	
	AP	PA
Non-responder	16	19
Responder	11	15

Fisher's Exact Test: Responder and Order
p-value = 1; Odds ratio (95% CI) = 1.1 (0.37 to 3.63)

Age

	Mean (SD) age in years
Non-responder	36.0 (9.27)
Responders	31.7 (7.16)

Welch Two Sample t-test: Age by Responder
t = 2.06, df = 58, p-value = 0.044

Sex

	Female	
	No	Yes
Non-responder	10	25
Responder	9	17

Fisher's Exact Test: Responder and Female
p-value = 0.78; Odds ratio (95% CI) = 0.8 (0.22 to 2.61)

CD4 T-cell count

	Median (IQR) CD4 T-cell count
Non-responder	450 (350 to 615)
Responder	435 (317 to 569)

Wilcoxon rank sum test: CD4 by Responder
W = 431.5; p-value = 0.52

Employment

	Employed	
	No	Yes
Non-responder	1	34
Responder	3	23

Fisher's Exact Test: Responder and Employed
p-value = 0.30; Odds ratio (95% CI) = 0.2 (0.004 to 3.08)

Education
(≥ 9 years of education)

	Education ≥ 9 years	
	No	Yes
Non-responder	4	30
Responder	3	22

Fisher's Exact Test: Responder and Education ≥ 9 years
p-value = 1; Odds ratio (95% CI) = 1.0 (0.15 to 7.36)

Symptoms: burning

	Burning	
	No	Yes
Non.responder	7	28
Responder	7	19

Fisher's Exact Test: Responder and Burning
p-value = 0.55; Odds ratio (95% CI) = 0.7 (0.17 to 2.69)

Symptom: painful cold

	Painful cold	
	No	Yes
Non-responder	10	25
Responder	5	21

Fisher's Exact Test: Responder and Painful cold
p-value = 0.55; Odds ratio (95% CI) = 1.7 (0.43 to 7.24)

Symptom: electric shocks

	Electric shocks	
	No	Yes
Non-responder	8	27
Responder	6	20

Fisher's Exact Test: Responder and Electric shocks
p-value = 1; Odds ratio (95% CI) = 0.9 (0.25 to 4.05)

Symptom: tingling

	Tingling	
	No	Yes
Non-responder	2	33
Responder	2	24

Fisher's Exact Test: Responder and Tingling
p-value = 1; Odds ratio (95% CI) = 0.7 (0.05 to 10.75)

Symptom: pins and needles

	Pins and needles	
	No	Yes
Non-responder	3	32
Responder	0	26

Fisher's Exact Test: Responder and Pins and needles
p-value = 0.25; ; Odds ratio (95% CI) = inf (0.31 to inf)

Symptoms: numbness

	Numbness	
	No	Yes
Non-responder	3	32
Responder	3	23

Fisher's Exact Test: Responder and Numbness
p-value = 1; Odds ratio (95% CI) = 0.7 (0.09 to 5.89)

Symptoms: itching

	Itching	
	No	Yes
Non-responder	13	22
Responder	4	22

Fisher's Exact Test: Responder and Itching
p-value = 0.08; Odds ratio (95% CI) = 3.2 (0.82 to 15.57)

Signs: touch hypoaesthesia

	Touch.hypoesthesia	
	No	Yes
Non-responder	33	2
Responder	25	1

Fisher's Exact Test: Responder and Touch hypoaesthesia
p-value = 1; Odds ratio (95% CI) = 0.7 (0.01 to 13.44)

Signs: pin-prick hypoaesthesia

	Pin-prick hypoaesthesia	
	No	Yes
Non-responder	18	17
Responder	10	16

Fisher's Exact Test: Responder and Pin-prick hypoaesthesia
p-value = 0.44; Odds ratio (95% CI) = 1.7 (0.54 to 5.42)

Signs: brush allodynia

	Brush.allodynia	
	No	Yes
Non-responder	26	9
Responder	19	7

Fisher's Exact Test: Responder and Brush allodynia
p-value = 1; Odds ratio (95% CI) = 1.1 (0.28 to 3.89)
