# Supplementary material for: Randomized, Double-Blind, Crossover Trial of Amitriptyline for Analgesia in Painful HIV-Associated Sensory Neuropathy
Source: PLoS One. 2015 May 14;10(5):e0126297. doi: 10.1371/journal.pone.0126297 (PMC4431817; doi:10.1371/journal.pone.0126297)

S4 Data. ANOVA summary and data plot for "average pain in the last 3 days" in ARV-users (intention-to-treat cohort: n = 62)

Between group

	Df	Sum Sq	Mean Sq	F value	Pr(>F)
Order	1	1.0	0.998	0.071	0.79

Within group

	Df	Sum Sq	Mean Sq	F value	Pr(>F)
Period	1	68.0	67.95	31.530	5.02e-08 ***
Time	2	56.1	28.04	13.011	4.11e-06 ***
Treatment	1	0.3	0.29	0.136	0.712

Significance codes: '***' 0.001, '**' 0.01, '*' 0.05

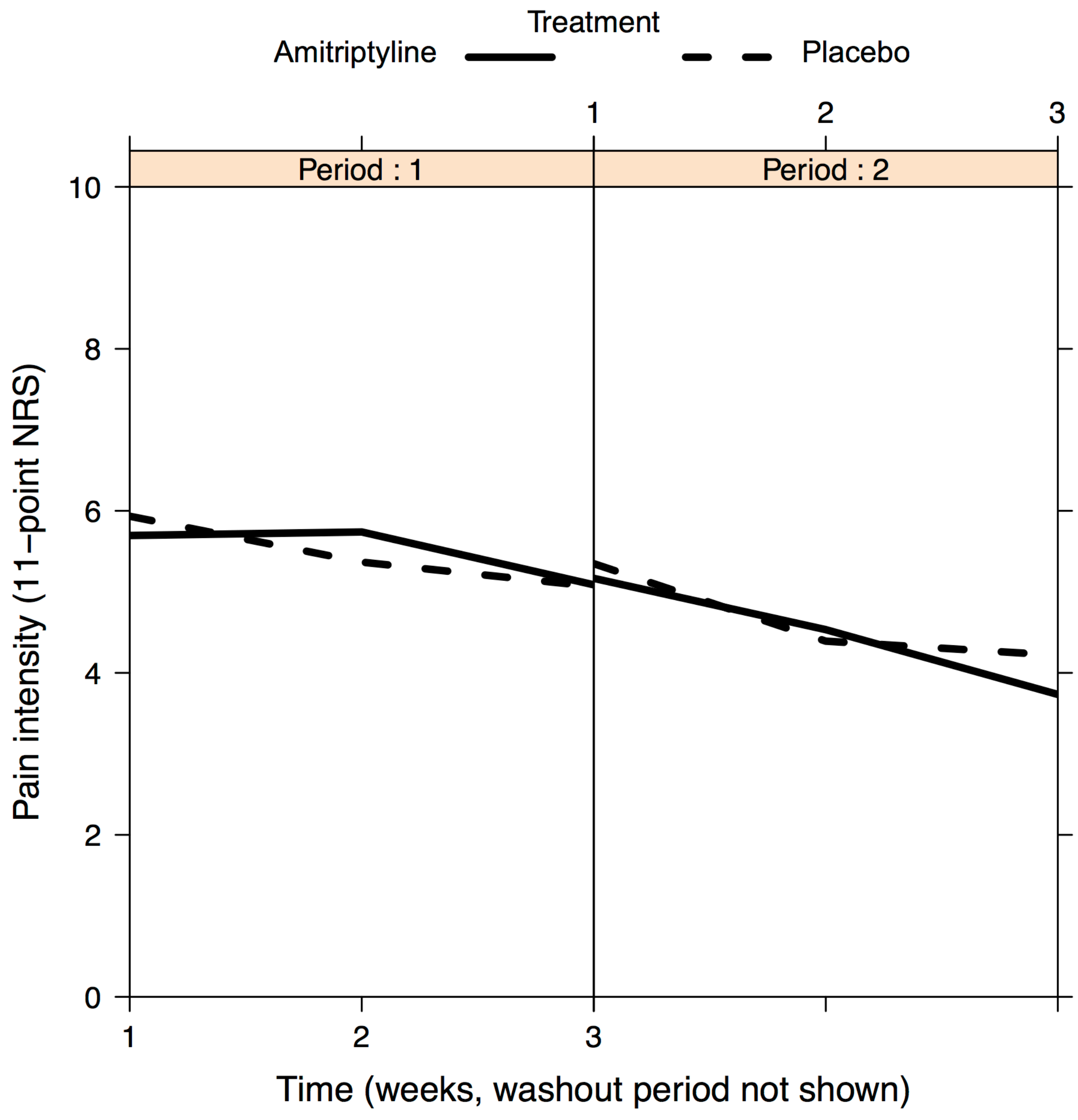

Supplement: S4 Data — (PDF) [file pone.0126297.s005.pdf]
