# Supplementary material for: Randomized, Double-Blind, Crossover Trial of Amitriptyline for Analgesia in Painful HIV-Associated Sensory Neuropathy
Source: PLoS One. 2015 May 14;10(5):e0126297. doi: 10.1371/journal.pone.0126297 (PMC4431817; doi:10.1371/journal.pone.0126297)

S5 Data. ANOVA summary and data plot for “average pain in the last 3 days” in ARV-naïve participants (intention-to-treat cohort: n = 62)

Between group

	Df	Sum Sq	Mean Sq	F value	Pr(>F)
Order	1	8.7	8.698	0.954	0.333

Within group

	Df	Sum Sq	Mean Sq	F value	Pr(>F)
Period	1	212.7	212.68	112.636	< 2e-16 ***
Time	2	138.2	69.12	36.608	5.88e-15 ***
Treatment	1	0.9	0.89	0.471	0.493

Significance codes: '***' 0.001, '**' 0.01, '*' 0.05

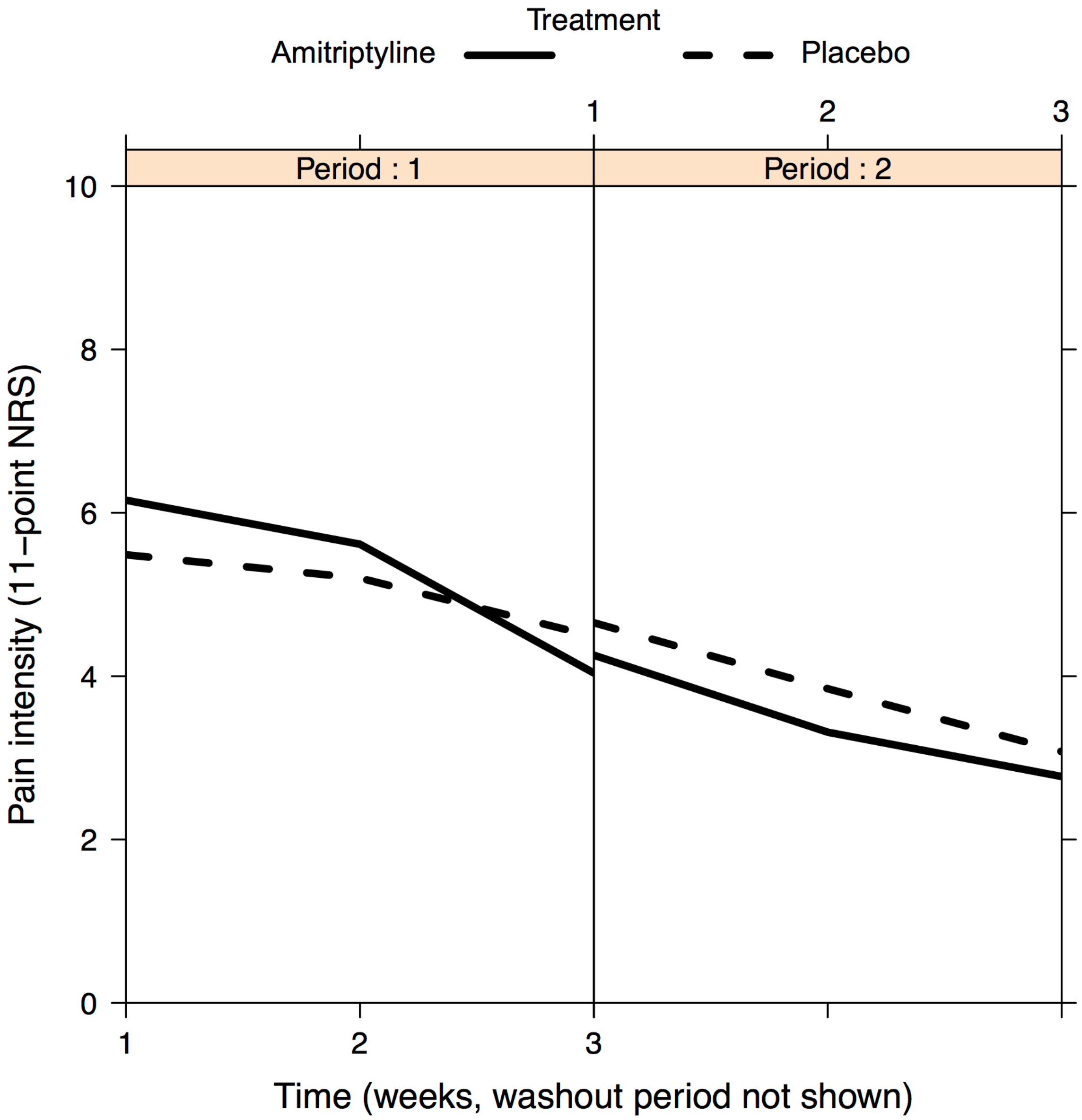

Supplement: S5 Data — (PDF) [file pone.0126297.s006.pdf]
