# Supplementary material for: Randomized, Double-Blind, Crossover Trial of Amitriptyline for Analgesia in Painful HIV-Associated Sensory Neuropathy
Source: PLoS One. 2015 May 14;10(5):e0126297. doi: 10.1371/journal.pone.0126297 (PMC4431817; doi:10.1371/journal.pone.0126297)
Supplement: S1 Protocol — (PDF) [file pone.0126297.s007.pdf]

Protocol:

A double blind randomised controlled trial to assess the effectiveness of Amitriptyline compared to placebo in the management of moderate to severe HIV related peripheral

Table of Contents

Abbreviations and acronyms 3
Trial abstract 3
Study Summary 3
Background 4
Rationale 7
Product information 7
Methodological issues 8
Population 9
Hypothesis 9
Primary outcome measures 10
Secondary outcome measures 10
Trial design 10
Study sites 10
Methods 10
Sample size calculations 10
Randomisation 10
Inclusion and exclusion criteria 11
Study procedures 12
Adverse event guidelines 17
Retention strategies 17
Visit windows 17
Unscheduled visits, withdrawal from study 17
Recognition and procedures for non-study symptoms and concerns 17
Ethical and regulatory considerations 20
Ethics review 20
Regulatory issues 22
Monitoring and DSMB 22
Data collection management and analysis 22
Dissemination 23
Logistical issues 23
Appendices 23

Abbreviations and acronyms

AE	Adverse event
ARV therapy	Antiretroviral therapy
DSN	Distal sensory neuropathy
DSMB	Data & safety monitoring board
EMEA	European Medicines Agency
EML	Essential Medicines list
HIV	Human immune deficiency virus
MCC	Medicines Control Council of South Africa
RCT	Randomised controlled trials
SA NDOH	South African National Department of Health
SAE	Serious Adverse Event
SOP	Standard operating procedure
WHO	World Health Organisation
Wits	University of the Witwatersrand

Trial abstract

A double blind randomised controlled trial to assess the effectiveness of amitriptyline compared to placebo in the management of moderate to severe HIV- related peripheral neuropathy. A cross over design will be used and 124 adult male and female volunteer participants with moderate to severe HIV related neuropathic pain will be followed up for a total of 15 weeks. Sixty two participants will be clinically and virologically stable on ARV drugs and 62 will not be in ARV therapy or intending to start in the next four months.
In the first six weeks participants will be given either amitriptyline or identical looking placebo titrated to either effect or a maximum dose of 150mg. Then following a three week ‘washout’ period, participants will be ‘crossed-over’ to the other arm and titrated to either effect or a maximum dose for 6 weeks. Pain intensity will be measured on a daily basis and recorded into a pain diary by the participant and regular telephonic contact will be maintained between the study nurse and the participants. Pain intensity using tools validated for neuropathic pain, as well as other data will be collected at face to face interviews at each of the 5 scheduled follow up visit. Data collected will include information on adverse events, physical functioning and quality of life.

Study Summary

Study purpose: to assess the effectiveness of Amitriptyline compared to placebo in the management of moderate to severe HIV- related peripheral neuropathy in a population using ARV and not using ARV therapy

Study design: A double blind randomised controlled trial using a cross over design

Sample Size: 124 participants; 62 on ARV therapy and 62 not on ARV therapy followed up for a total of 15 weeks

Study population: adult men and women with documented HIV infection and moderate to severe HIV related distal sensory neuropathy

Visit schedule: An information session with recruitment, followed by a screening visit and an enrolment visit. After enrolment there will be five scheduled visits and weekly telephonic follow-up if written consent provided for that. Optional visits to the Palliative care centres may be done at any time during office hours for concerns, advice or further information. After hours telephonic contact will be available.

Study duration: the total duration of the study is 14 months. Three months for start up, training and preparation, enrolment closes after 6 months; the last participant exiting at month 11, and three months analysis and write up.

Primary objective: A measurement of the difference in pain intensity at the beginning and end of each 6 week treatment period.

Secondary objective: These will be adverse side effects of treatment, minimum effective doses, maximal tolerated doses, mood and effect on activities of daily living.

Study sites: Participants will be recruited from the Nthabiseng HIV Clinic at the Chris Hani Baragwanath Hospital, the Helen Joseph Hospital HIV clinic, and the palliative care clinic and outreach services.

Regulatory and monitoring: The Medicines Control Council (MCC) approval is not required since the drug is being used for indicated purposes. The MCC will be notified. The trial will be registered with the SA National Trials Register, and the Cochrane trials register. Ethical approval will be sought from the Wits REC and regular progress reports submitted as well as SAE, AEs and protocol violations. A study DSMB will be constituted who will perform an interim analysis as well as monitor the trial for safety and protocol violations.

Ethical issues: Placebo is justified on the basis that there is no comparator established nationally or internationally as yet. A clinician's advice will be sought for those identified with severe DSN pain which may be ARV related and in whose interest a change in ARV regime may be warranted instead of pain relief. Patients *in extremis* in pain will not be eligible and provision for rescue treatment for all participants will be available.

Background:

Neuropathic pain in HIV

Moderate to severe neuropathic pain will be experienced by a considerable proportion of people with HIV, adversely affecting their activities of daily living, mood and sleep, fatigue and may have an impact on physical or social functioning. It can be debilitating to the point of severe mobility restriction. Unlike nociceptive pain neuropathic pain has no protective role but the exact mechanisms involved in the generation of various neuropathic pains remain elusive. Neuropathic pains in HIV represent heterogeneous clinical conditions which cannot be explained by a single mechanism. Clinical conditions which relate to HIV infection include inflammatory demyelinating polyneuropathy, mononeuritis multiplex, progressive radiculopathy. Aetiologies vary and are thought to arise from: ARV therapy especially with dideoxynucleosides (stavudine/D4T, a first line treatment in South Africa); damage from the virus itself; vitamin B12 deficiency; those caused by opportunistic infections, for example a post-herpetic neuralgia, treatment with other drugs such as anti TB drugs.

This trial will look at the effectiveness of treatment for the most common form of HIV related neuropathy, distal sensory neuropathy (DSN). Studies have estimated that distal sensory neuropathy (DSN) is present in up to 40%-50% of people with HIV on antiretroviral therapy (ARV) and 90-100% of those with DSN have pain(1). This has not changed with improvements in ARV therapy. Increased survival with ARV therapy has led to an increase in the prevalence of DSN. Risk factors for the development of DSN include advancing age, higher peak plasma viral load levels, exposure to didanosine drugs and protease inhibitors. (2) There are two sub-types of DSN: the type solely associated with HIV infection and the type associated with ARV therapy, sometimes referred to as acute toxic neuropathy (2). Work at the palliative care unit at Wits has demonstrated effective pain and symptom relief, using a validated tool (Wits ethics number M060366) in patients with HIV. However a subset of patients has not responded to current pain treatment protocols. It is postulated that these patients with 'difficult' pain are those with neuropathic pains. Effective pain management in HIV may improve adherence to ARV therapy, especially when the pain is caused by the ARV therapy itself.

What is known about treating HIV related DSN

Clinical trials have shown that treatments effective in other disease states cannot be transferred to HIV DSN(2). Thus the effective use of drugs such as amitriptyline or other tricyclics, carbamazepine, lamotrogine, gabapentin , or other anti –convulsants ; opioids or indeed drug combinations in other disease states do not necessarily mean effectiveness in HIV DSN. The results of negative trials in HIV DSN include assessment of acupuncture, mexilteline, peptide T. Other RCT results are listed below.

Results of randomised controlled trials carried out in this area:

Drug	Comment	effectiveness
Lidocaine	Small trial	none
Gabapentin	Small trial (3)	Could be effective
Amitriptyline	2 trials, see details below	Not effective
lamotrigine	Conflicting evidence(4;5)	One trial yes, another no

Also drugs which may have interaction with ARVs have been excluded for consideration, for example sodium valproate. New treatments, including an open labelled study which suggests that capsaisin is effective, are on the horizon but treatment costs and future drug availability and timelines for evidence are uncertain. There are no non-drug interventions which have been shown to be effective for HIV –DSN.

Current guidelines internationally recommend either gabapentin, amitriptyline or lamotrigine. Nationally amitriptyline is recommended. None of these are based on high grade evidence. There have been two RCTs evaluating amitriptyline for pain due to HIV related distal sensory neuropathy(6;7). One by Shlay and co-workers in 1998 compared amitriptyline to acupuncture and matching placebo. The second trial by Keiburtz et. al compared amitriptyline to mexiletine and matching placebo. Both showed no effect for amitriptyline. However we are proposing that it is important to conduct this trial for reasons which are elucidated below;

- The study populations in the two trials are different to the ones we propose.
    - White (70-82%) male (96%) with mild to severe pain

- In the light of newer work on the role of response variation with genotype to the development of DSN, trials are warranted in a South African population (8;9) (10)
- Both trials included participants with all grades of pain, and in both trials the mean for baseline pain were mild or moderate. The European Medicines Agency recommend that trials are conducted in people with moderated to severe pain, since in a mild pain population a high response to placebo can be expected(11)
- Both trials had limitations in terms of enrolment numbers not reaching calculated samples sizes or original trial design
- One trial found a trend for improvement of pain in the amitriptyline group

Finally amitriptyline is widely used for this condition in South Africa. It is the drug of choice for many treatment protocols throughout the country. Personal communication with Aspen Pharmaceutical indicates that the bulk of their brand (trepiline) is used for DSN. In addition it is recommended in the South African palliative care national guidelines, by the SA HIV Clinicians society and the Foundation for Professional Development for HIV related DSN. Thus it is important to support or refute existing evidence in a population that is clinically relevant to South Africa.

Implications for South Africa of this trial generating new evidence to support or refute pre-existing evidence

With the growing number of people with HIV related DSN and its impact on social and physical functioning of individuals, it is important do this trial. Cost and availability of drugs is also important to consider. One recent paper estimated that one year of treatment of painful neuropathy in the USA cost $17,000 per person per year(12). In addition drugs found to be effective in this condition are expensive. For example lamotrogine is currently R16 per day compared to R 0.30 per day for amitriptyline, according to the Chris Hani Hospital formulary of 2006. In addition, gabapentin is not widely available at present, although that may change in the near future as a local generic version has been registered. Amitriptyline is on the WHO and SA EML, and is relatively cheap and widely available in primary health care clinics as well as hospitals.

Clinical issues of importance when considering drug of choice

Any drug candidate for treatment of DSN should not have interaction with ARV therapy, either documented or theoretical. For example carbamazepine is not recommended for use with ARV therapy because of drug interaction with nucleosides(13). The SA DOH first and second line recommendations or commonly used drugs by people with HIV such as contraceptive pills, fluconazole do not have listed drug interactions with amitriptyline. Other trials have not found that amitriptyline adversely affects viral load or CD4 counts, or any other blood marker for safety. Therefore it is not considered necessary in this trial to measure viral load. The side effects of the drug should be considered. The Schlay trial and the Kieburtz trials noted that amitriptyline was well tolerated at dosages of up to 100mg per day(6;7). This concurs with local experiences. The pill burden, drug formulation and side effects should also be considered and are proposed to be acceptable for amitriptyline. Since people with HIV often experience multiple pains occurring concurrently, a screening tool validated for HIV related DSN will be used and pain treatments will be documented. Primary

outcome measures will not be changes in mood, sleep or quality of life, since these may be attributable to treatments of other pains.

Rationale

Pathophysiology of NPs

Although a clear separation between peripheral and central neuropathic pain has been established, peripheral nerve damage has been shown to lead to neuroplastic changes in the central nervous system with somatosensory implications.
DSN in HIV are poorly understood but it is agreed that several pathopysiological mechanisms exist. Neuropathy is charactersied by pain related phenomena such as allodynia, hyperalgesia , dysaethesia and paraesthesia. Sensory dysfunction, for example numbness, pins and needles or loss of vibration sense may also be present. Autonomic responses resulting in mottling of skin may also be seen. Pains are often spontaneous, or evoked by non-noxious stimuli, may be persistent and fluctuating or periodic. Incidence and severity is related to viral load(14). Untreated DSN leads to increasing area of pain, allodynia, and hyperesthesia, as well as sensory loss.

Product information

Amitriptlyine has antidepressant properties belonging to the tricyclic group. Indicated use is for management of depressive illness, nocturnal enuresis, and pain relief in chronic pain syndromes. It is well absorbed orally, has a half life of between 10 and 50 hours (average 16 hours). It is extensively metabolized in the liver, involving microsomal hepatic enzymes and eliminated over several days, primarily in the urine.

Contraindications are early post-myocardial infarct period and heart block. Use with caution is recommended in patients with: hyperthyroidism, or on thyroxine therapy; arrhythmias; epilepsy; prostatic enlargement; closed angle glaucoma or impaired liver function. Amitriptyline has no drug interactions with ARV therapy. Drug interactions include those with antihistamines, antipsychotics, anti-cholinergic type anti parkinsonian agents, cimetidine, fluoxetine, paroxetine, steroids, including oral contraceptives and MAOI drugs. It is not to be used in pregnancy.

Amitriptyline for HIV related DSN

The postulated mechanism of action for HIV related DSN is a balanced reuptake inhibition of both serotonin and noradrenalin(15).

Safety, tolerability and side effects and recommended doses: Studies have noted no significant differences between placebo treated groups and amitriptyline in when compared using ECGs or laboratory tests for viral load, CD4 count, liver function(6;16).

Dose regime, other drug trials have shown effect at 10mg- 150mg. Our regime will be titrated to effect in the following manner: 25mg-50mg-75mg-100mg-150mg, then to stop.
Route- oral route with a frequency: once daily at night to mitigate possible side effects of drowsiness and postural hypotension.

Methodological issues

Placebo

An inactive placebo will be used. It will be identical looking to the active drug and be manufactured according to Good Manufacturers' Practice (GMP guidelines and standard operating procedures). The use of a placebo rather than proven treatment is recommended here because of the lack of known effective and available treatments. There is no treatment standard in South African except amitriptyline, so no choice for a comparator. The European medicines agency has developed guidelines for clinical development of new medicinal products for neuropathy and they include the recommendation that in the light of poor evidence for a comparator to use a placebo controlled trial. Ethical issues in using placebo in pain trials are discussed below. Inactive placebo is used rather than active since in the investigators experience and from the literature, the low dose used (25-75mg), does not seem to cause common or significant side effects that may differentiate active drug from placebo. The placebo effect in pain studies is well documented and its magnitude has been taken into consideration in calculating the sample size.

Cross over design

A cross over design is proposed for the following reasons: HIV has a complex disease course, and it is acknowledged that there may be many different mechanisms for the DSN so it is desirable if the participant acts as their own control. It is considered that the disease course will not significantly deteriorate in the follow up period of 15 weeks, although the baseline pain intensity will be measured at the start of each treatment intervention. It is anticipated that the follow up period is not too long to see a significant attrition rate. There will be two sub-groups: those stable on ARV with DSN and those not on ARV drugs. Secondly, the disease process is fairly stable over the proposed 14 weeks and the survival times for most participants will be more than proposed trial period.
Washout period: The SAMF 8 cites a plasma t1/2 of 10-50 hours, but also notes that the nortriptyline metabolite is active. The rule of thumb would be 5 half-lives, but in this case, you'd need to track down the metabolite's elimination. (Handbook of Basic Pharmacokinetics including Clinical Applications (6th edition). Ritschel WA and Kearns GL. APHA, Washington, 2004) gives a t1/2 for nortriptyline of 14-90 hours. On that basis, you'd be looking at a wash-out of at least 450 hours, or 18.75 days. Personal correspondence Prof Andy Gray

Measuring pain intensity

Pain is a subjective experience so self reported pain scores on a visual analogue scale or 11 point Likert numerical rating scale is recommended. A tool (Brief peripheral neuropathy screening tool, BPNS) which has been validated for screening for HIV related DSN will be used(17) along with another tool the DN4 which is validated for DSN. Electrophysiological variables and sensory evaluation do not correlate with the severity of DSN, so will not be used as measures of primary efficacy. The baseline pain score and subsequent scores will be measures using the 11 point Likert numerical rating scale, plus the DN4 and the BPNS tool.

Population

ARV and no ARV

The possibility of effect for subpopulations will be factored into the trial design, with a sample size calculated to stratify got two groups , those stable on ARV and those not on ARV drugs. Changes in ARV regime may interfere with DSN, so become confounding factors and difficult to interpret. Thus only those stable on ARV drugs for three or more months will be considered eligible.

Moderate to severe pain

The European Medical Agency recommends that research is conducted on participants with severe or moderate neuropathic pain only (11).

Genetic consideration

Work in Australia and at Wits University has indicated that genetic factor may be a significant factor in the development of ARV related ATN and response to treatments. Clinical trials have included few black African participants with DSN(18;19).

Gender considerations

Neuroplasticity responses may be different in women, as may the metabolism of drugs(19). In SA more women are infected with HIV, yet studies in DSN have been mainly in men.

All conditions which are likely to cause confounding will be excluded from the study, together with conditions which are likely to cause drug interactions.

- Severe pain from DSN caused by ARV drugs which warrants a change in regime
- Already taking amitriptyline drug, or has taken the drugs at least three weeks prior
- Limb amputation
- Kaposi sarcoma of the lower limbs
- Current post- herpetic neuralgia or herpes zoster
- Pregnancy or intention to fall pregnant
- TB treatment
- Malignancy not related to HIV
- Major psychiatric disorders including mania or epilepsy
- Clinically significant renal failure
- Diabetic neuropathy
- Clinically significant liver failure or past history as defined by encephalopathy, oedema and jaundice
- Patient in extreme pain or exhausted
- Participating on another trial or study
- MAOI, other tricyclic antidepressants or anti-epileptic drugs
- Recent myocardial infarction, arrhythmias, heart block
- History of urinary retention, urinary hesitancy or closed angle glaucoma

Hypothesis

Amitriptyline is superior to placebo in the management of moderate to severe HIV related peripheral neuropathy.

Primary outcome measures

Self reported pain intensity measurement is the primary outcome measure.

Secondary outcome measures

These will be adverse side effects of treatment, minimum efficacious doses, maximal tolerated doses, mood and effect on activities of daily living

Trial design

A placebo controlled double-blind crossover group randomised controlled trial will be conducted. The placebo will be a non-active substance.

Study sites

Participants will be recruited from the Nthabiseng HIV Clinic at the CHBH, who have more than 5000 patients on their books, the HJH HIV clinic (more than 7000 patients on ARV therapy), and the palliative care clinic and outreach services. The clinics are Government ARV accredited sites, and see patients who are not yet eligible for ARV therapy. The palliative care clinic and outreach services see patients who are on ARV therapy and those who have declined, no longer on the ARV. The potential participants will be recruited by a non-clinic staff member. Initially recruitment will occur at the CHB hospital. If required then recruitment will take place at the HJH. All the clinics and services are at the hospital site or hospital outreach. The interviews, examinations and data processing will take place at the CHB palliative care centre for excellence.

METHODS

Sample size calculations

These have been calculated by a statistician, in the Wits School of Public Health.

The sample size will be determined with the following variables considered:
90% power, $\alpha=0.05$, effect is difference of 2 on a 0-10 scale; variance is 2SD. Variability of the distribution of the population was estimated from existing local clinical data and from previous estimates in the literature. The size of the placebo effect was estimated after considering the literature. For a cross-over design 46 participants will be required. Assuming two distinct sub-populations, those stable on ARV therapy and those not on ARV therapy and a loss to follow-up of ~10%.

Enrolment target is 124 participants: 62 on ARV therapy and 62 not on ARV therapy

Randomisation

The randomisation allocation provided by the manufacturers of the placebo and active product. Participants will receive either regime A or regime B in a random manner. One regime will contain 6 weeks of active substance, followed by three weeks washout, then with 6 weeks of placebo, the other will contain 6 weeks of placebo followed by three weeks washout, then 6 weeks of active substance. Patients will be randomly allocated via a concealed allocation; the randomisation will be generated by block randomisation methods.

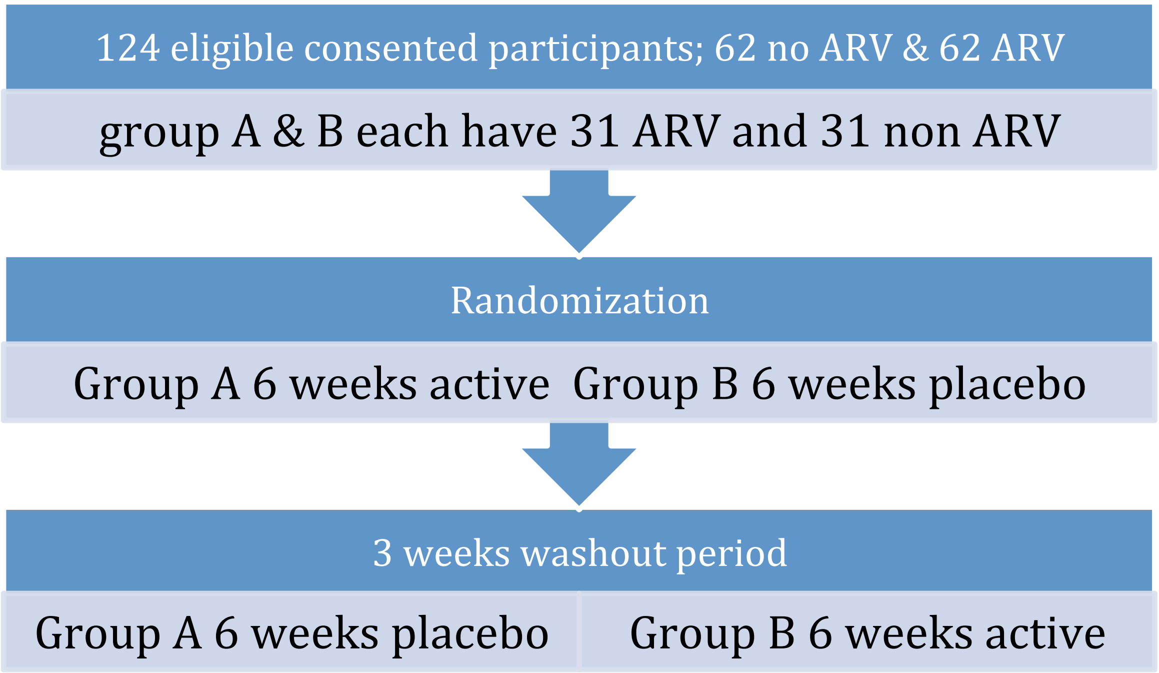

Inclusion and exclusion criteria:

The following criterion will be used to select patients:

1. Confirmed and documented HIV positive status, either on Arv therapy or not on ARV therapy
2. Diagnosed with moderate to severe peripheral neuropathy using the BPNS and DN4 screening tools and scoring >/= 4 on an 11 point Likert scale visual analogue scale.
3. Age above 18 on the date of recruitment
4. Ability to mentally and physically understand to make informed choice to participate in the study

All patients who fit the above criterion will be eligible for participation.

Exclusion Criteria

All conditions which are likely to cause confounding will be excluded from the study, together with conditions which are likely to cause drug interactions.

- Severe pain from DSN caused by ARV drugs which warrants a change in regime
- Already taking amitriptyline drug, or has taken the drugs at least three weeks prior
- Limb amputation
- Kaposi sarcoma of the lower limbs
- Current post- herpetic neuralgia or herpes zoster
- Pregnancy or intention to fall pregnant
- TB treatment
- Malignancy not related to HIV
- Major psychiatric disorders including mania or epilepsy
- Clinically significant renal failure
- Diabetic neuropathy
- Clinically significant liver failure or past history as defined by encephalopathy, oedema and jaundice

- Patient in extreme pain or exhausted
- Participating on another trial or study
- MAOI, other tricyclic antidepressants or anti-epileptic drugs
- Recent myocardial infarction, arrhythmias, heart block
- History of urinary retention, urinary hesitancy or closed angle glaucoma

Study procedures

Recruitment method

Patients will be recruited from Chris Hani Baragwanath Hospital HIV clinic, Helen Joseph HIV Clinic and the Palliative care community outreach unit and out-patients clinic. The patients will be actively recruited by the study doctor and nurse, and screened for eligibility. In addition clinic staff will be informed of the study and be able to invite patients who show interest to find out more from the study team. Posters will be displayed in the clinics providing information on the study.

Screening procedure

Patients agreeing to participate will undergo screening. The following will happen during this visit:

1. Information session on the study. Posters and group recruitments will be supported by one-to one information sessions
2. Assess eligibility for study
3. Written consent will be obtained to have a short eligibility questionnaire administered and a test for ankle reflexes and vibration sense. See appendices
4. Inform participant of eligibility status then either thank participant for involvement in screening and referral to palliative care or other services if appropriate. Or if eligible invite them to information session in the study now or at a time convenient to them.

Baseline procedure

The following is the procedure for the baseline visit:

1. Detailed information session on the study
2. Time to consider participation and invite questions and clarification
3. If agrees to join study then administer written informed consent. If not or changes mind then thank the participant for their time
4. For those enrolled collection of demographic data(Appendix 3)
5. Collection of clinical data(Appendix 4)
6. Assessment of peripheral neuropathy(Appendix 5);
7. Review eligibility criteria and if still eligible and consenting then randomization will occur
8. Participant will be given arm treatment, pain diary and written and verbal instructions. They will receive an airtime voucher or telephone card for R28 per month for the duration of the study and a telephone number to call should they need to call the study team

Follow up procedures: Day 4-day 15

At home: one tablet will be taken at night for three nights. On the third day the study nurse will call to enquire as to adverse effects, pain score and any other problems, and advice on the next dosage. If the pain has abated significantly the dose will be left at one tablet at

night. If not, the dose will be increased to two tablets at night for three nights. The same procedure will occur until a maximum of 6 tablets at night has been reached. The participant will continue to take this dose for 4 weeks. A weekly telephone call will be conducted by the study nurse.

Day 3	The study nurse will call to enquire as to adverse effects, pain score and any other problems, and advice on the next dosage. If the pain has abated significantly the dose will be left at one tablet at night. If not, the dose will be increased by two tablets at night for three nights.
Day 7	The study nurse will call to enquire as to adverse effects, pain score and any other problems, and advice on the next dosage. If the pain has abated significantly the dose will be left at two tablets at night. If not, the dose will be increased to three tablets at night for three nights.
Day 11	The study nurse will call to enquire as to adverse effects, pain score and any other problems, and advice on the next dosage. If the pain has abated significantly the dose will be left at three tablets at night. If not, the dose will be increased to four tablets at night for three nights.
Day 13	The study nurse will call to enquire as to adverse effects, pain score and any other problems, and advice on the next dosage. If pain has abated significantly the dose will be left at four tablets at night. If not the dose will be increased to six tablets at night. I
Week 3 day 15	The study nurse will call to enquire as to adverse effects, pain score and any other problems, and advise to stay at the same dosage for the next 4 weeks
Visit 3.0 Week 3 day 21	Visit to study site. Check diary, questionnaire administered, medication check, discussion and information as required with participant, reimbursement, referral as required
Week 4 day 28	The study nurse will call to enquire as to adverse effects, pain score and any other problems, and advise to stay at the same dosage for the next 3 weeks

Week 5 day 35	The study nurse will call to enquire as to adverse effects, pain score and any other problems, and advise to stay at the same dosage for the next 2 weeks
Visit 6.0 Week 6 day 42	1. Visit to study site Check diary, questionnaire administered, medication check, discussion and information as required with participant, reimbursement, referral as required Collection of demographic data(Appendix I)<br>2. Collection of clinical data(Appendix II)<br>3. Assessment of peripheral neuropathy and other symptoms(Appendix III); if present refer to Palliative care<br>4. Assessment of coexisting pain medication
Week 7 day 49	The study nurse will call to enquire as to adverse effects, pain score and any other problems
Week 8 day 56	The study nurse will call to enquire as to adverse effects, pain score and any other problems
Visit 9.0 Week 9 day 63	Visit to study site Check diary, questionnaire administered, medication check, discussion and information as required with participant, reimbursement, referral as required. Patient to receive new drug pack
Week 10 Day 66	The study nurse will call to enquire as to adverse effects, pain score and any other problems, and advice on the next dosage. If the pain has abated significantly the dose will be left at one tablet at night. If not, the dose will be increased to two tablets at night for three nights.
Day 69	The study nurse will call to enquire as to adverse effects, pain score and any other problems, and advice on the next dosage. If the pain has abated significantly the dose will be left at two tablet at night. If not, the dose will be increased to three tablets at night for three nights.

Week 11 day 72	The study nurse will call to enquire as to adverse effects, pain score and any other problems, and advice on the next dosage. If the pain has abated significantly the dose will be left at three tablets at night. If not, the dose will be increased to four tablets at night for three nights.
Day 75	The study nurse will call to enquire as to adverse effects, pain score and any other problems, If not, the dose will be increased to six tablets at night.
Day 78	The study nurse will call to enquire as to adverse effects, pain score and any other problems, and advice to stay on the same dosage for the next 4 weeks.
Visit 12.0 Week 12 day 82	5. Visit to study site Check diary, questionnaire administered, medication check, discussion and information as required with participant, reimbursement, referral as required Collection of demographic data(Appendix I)<br>6. Collection of clinical data(Appendix II)<br>7. Assessment of peripheral neuropathy and other symptoms(Appendix III); if present refer to Palliative care<br>8. Assessment of coexisting pain medication
Day 91	The study nurse will call to enquire as to adverse effects, pain score and any other problems, and advice to stay on the same dosage for the next 2 weeks
Day 98	The study nurse will call to enquire as to adverse effects, pain score and any other problems, and advice to stay on the same dosage for the next 1 weeks
Exit procedure Week 15 is the exit visit and visit to study site.	Check diary, questionnaire administered, medication check, pain intensity score, clinical examination and discussion and information as required with participant, reimbursement, referral as required. Information on study results feedback session

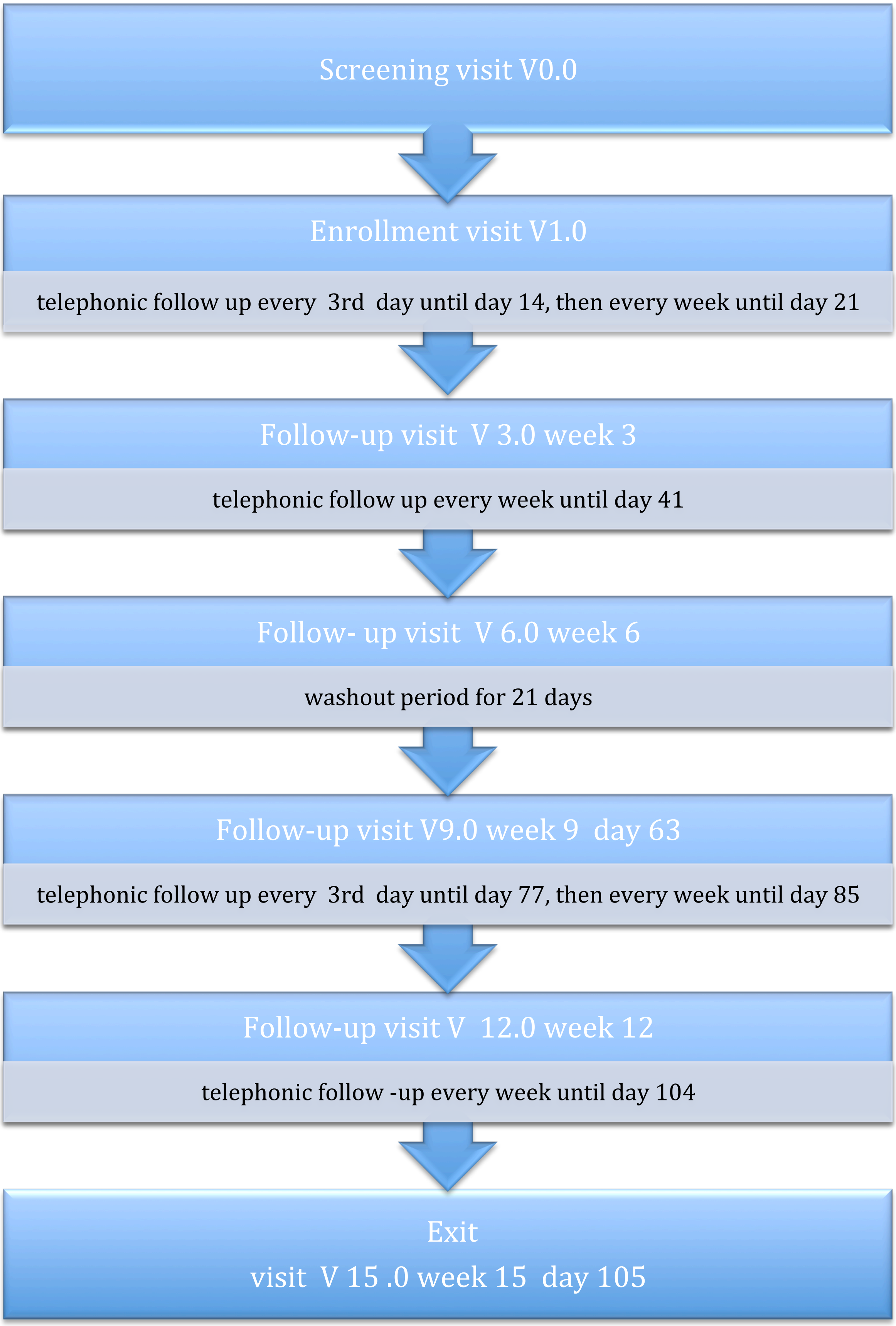
Screening visit V0.0
Enrollment visit V1.0
telephonic follow up every 3rd day until day 14, then every week until day 21
Follow-up visit V 3.0 week 3
telephonic follow up every week until day 41
Follow- up visit V 6.0 week 6
washout period for 21 days
Follow-up visit V9.0 week 9 day 63
telephonic follow up every 3rd day until day 77, then every week until day 85
Follow-up visit V 12.0 week 12
telephonic follow -up every week until day 104
Exit
visit V 15 .0 week 15 day 105

Adverse event guidelines

Downloaded from http://www.witshealth.co.za/Portals/0/Ethics/POL-IEC-001.doc
An adverse events is defined as follows; any untoward medical occurrence that may be present during treatment with a medicine/intervention but which does not necessarily have causal relationship with this treatment.

Adverse drug reaction: a response to a medicine that is noxious and unintended. The causal relationship between the reaction and the intervention may be a reasonable possibility.

Unexpected adverse reaction: one in which the nature, specificity, severity or outcome is not consistent with the product information.

Serious adverse event: an untoward medical occurrence that results in death, is life-threatening, requires patient hospitalisation or prolongation of existing hospitalisation, results in persistent or significant disability/incapacity or is a congenital anomaly /birth defect.

All these shall be reported as per Wits HREC requirements within the expected timeframe and in the correct format.

Unblinding of treatment arms will be done if required.

Retention strategies

Regular conversations will occur with participants on the study, building a rapport with participants. Appointments will be made trying to accommodate scheduled clinic visits. An SMS will be sent if the participant allows to remind them of upcoming appointment. Home visits is consented for will be done to remind those who have not come for their visits within three working days of the missed visit. Regular reports will be provided to the study team in the retention rates and those with missed visits. Reimbursement will be provided for incoming journey the visit before to facilitate travel.

Visit windows

A window period of 3 days will be allowed either side of the appointment date. Patients will be reminded of the upcoming appointment 2 days before.

Unscheduled visits, withdrawal from study

Extra visits will be allowed for adverse event. They will be coded thus; if in week 3, V 3.1. Withdrawal from the study shall be immediately effected on request, a form in reasons for withdrawal shall be filled in and another either allowing or withdrawing permission for utilisation of existing data.

Recognition and procedures for non-study symptoms and concerns

Should the study staff be alerted to concerns and symptoms not related to the study, they will act in the following manner, whilst recognising the importance of maintaining confidentiality as far as in the best interest of the participant:

Life-threatening conditions or other emergencies: provide immediate clinical intervention as best possible, and then refer for emergency admission

Major clinical conditions – written (when appropriate) referral to appropriate services, consider admission

Minor clinical conditions - written (when appropriate) referral to appropriate services
Concerns – referral to appropriate services

Protocol violations
Based on the approved study protocol standard operating procedures will be written and kept in an accessible file, dated and updated for all study staff. Staff will sign off on each relevant SOP. Should any study procedures be violated, the PI will be immediately informed, and a written report submitted to the Wits Ethics committee and the DSMB, within two days. If the situation requires Wits HREC mentioned will be notified immediately. The study staff undertake to run the study to ethical practice, and according to GCP guidelines.

Guidelines for introducing amitriptyline
Patients in this group will be given amitriptylline according to the following protocol. The dose of the dug will not be affected by the weight of the patient.

Dose	Days
25mg od at night<br>increase the dose on next visit if pain persists	Day 0
50mg od at night<br>increase the dose on next visit if pain persist	Day 3
75mg od at night<br>increase the dose on next visit if pain persist	Day 7
100mg od at night	day 11
150 mg od at night	Day 15

Side effects
Side effects will be continually monitored throughout the study as part of symptom control. (Appendix IV)

**Amitriptyline**
Dry mouth
Sedation
Weight gain
Confusion
Dizziness
Palpitations
Constipation

Other

Patients who develop side effects will be assessed by a doctor. Patients assessed as having serious adverse events will be withdrawn from the study, and reasons recorded. The total period of the study will be 6 weeks from enrolment. After being enrolled, subjects will be monitored for pain relief, symptom control and quality of life by the nurse, after which an intervention will be put in place. Monitoring will be done weekly by a professional nurse. Intervention can be one of the following:

- Increase the dose, and continue with the trial
- Maintain the dose, and continue with the trial
- Stop the drug because of adverse events, and drop from the study

For those that have been found to be not effective, alternative treatment will be offered.

Detection and management of adverse events
An adverse event is defined as **any untoward medical occurrence in a trail participant who has been administered a pharmaceutical product.**

Serious adverse event is defined as a specific type of adverse event which result in the any of following:

- Death
- Life threatening
- Hospitalization, or causes prolonged hospitalization
- Significant disability or incapacity
- Causes congenital anomaly

All adverse and serious adverse events will be reported to the ethics committee by the principal investigator.

Quality control
All the doctors, nurses and pharmacists participating in the study will be trained on the specific neurologic examination required for patients. The investigator will be the overseer of the site

During each visit, data collected on the questionnaires will be checked for completion and verified. Information of the patients will be verified at each visit to ensure that it remains current. All the information for the trial will be locked up at the Wits Palliative care offices. Required files for a specific day will be retrieved for use on the specific day, and returned to the main office.

Data collection, management and analysis
All patients will be allocated a unique identifier that will also serve as their file number on site. All the information for the patient, laboratory results, and questionnaires from different visits, together with the signed informed consent will be kept in the patient's file. The files will be locked up in a cupboard with limited access control.

Double entry of data collected will be entered using Microsoft Access. R statistical package will be used analyse the data. Descriptive statistics will be used to describe the demographics and clinical characteristics of the population studied.
The primary outcome (pain relief between baseline and end of the study) will be compared for the

- significance of overall pain relief for repeated measures using univariate analysis of variance (ANOVA)
- Association of the drug and the level of pain relief between different subjects using ANOVA.

The number needed to treat (NNT) will be calculated to assess the effectiveness of the drugs, while the number needed to get severe side effects will be calculated an NNF. During analysis subjects will be adjusted according to exposure to neurotoxic drugs. Analysis will also be done using the intention to treat analysis, where all randomised subjects will be included in the analysis to reduce bias.

The number treated analysis will also be done looking only at those subjects who managed to reach the maximum dose of the drugs. This however is subject to selection bias as subjects who developed side effects and those who were lost to follow up will be excluded. The effect of the use of other pain medication in together with the intervention being tested will also be analysed.

The results will be adjusted for ART during analysis, as all patients including those already on ART will be included in the study.

Limitation of the study
It will not be possible to ascertain the aetiology of the neuropathy, as it could be due to HIV itself, the use of neurotoxic drugs, or HIV related opportunistic infections. Because of ethical issues, it is not possible to do a double blind study as a way of reducing bias, hence the open study.

The doctor prescribing medication will only be aware of the drugs being given to a particular patient after the patient has been randomized, thus selection bias will be reduced.

ETHICAL AND REGULATORY CONSIDERATIONS

Ethics review

The study shall design and conduct shall be in accordance with the following
Ethical approval will be sought from the University of the Witwatersrand ethics committee and from the South African medicines Control council. The trial will be preregistered on the South African drugs trial register and the Cochrane register. Permission will also be sought from CHBH and Helen Joseph hospital. Written informed consent will be sought from all patients. The information leaflet to patients will be verbally translated to isiZulu and Sesotho. The refusal to participate will not influence the access of patients to treatment, in particular peripheral neuropathic pain, in which case standard protocol for the site will be used. Patients will be treated for all pains and symptoms as per palliative care protocols. Referral to ARV services will be made as required. With palliative care services being the exception, studies have found that pain in HIV is under-diagnosed and undertreated. It has been

estimated that more than 95% of people with HIV do not receive adequate pain relief in South Africa.
Benefits to study: Pain in HIV is widely under-diagnosed and undertreated. Participants will receive a thorough assessment of their DSN, they will receive acknowledgement of their pain and an explanation as to the nature of their pain. In addition each one will receive a period of active drug which may or may not provide pain relief.

Risks to being on this study. The participant will risk experiencing unwanted side effects from this drug. Although the presence of these will be carefully monitored at regular intervals. Intense follow-up, the keeping of a pain diary will take some time of the participants day which may cause inconvenience.

There will be no incentive or inducement to join this study, although the participants will not be out of pocket and receive cash (R70) for transport and time spent, and a phone voucher (either cell phone or Telkom) to call the study should they need to.

Placebo-controlled: The ethics of a placebo controlled trial as opposed to head to head comparison may be debated. In the presence of increasing evidence of effective treatments it would be argued that a head to head comparison be used. However there is no evidence of an effective treatment for HIV related DSN. Thus a comparator would be hard to come by. Also in pain trials the placebo effect is well documented, making a conclusion more difficult.
Use of other pain relieving medication for other pains or as rescue treatment: Participants will be able to continue to use other pain relieving drugs that they may be having or to access ‘rescue’ treatment (either mild opiate or NSAID). If amitriptyline is the only feasible option, they will be removed from the study and offered amitriptyline. Use of rescue medication for NP will be allowed and documented, and its impact on the results will be taken in to account in the analysis.

Cross over design is feasible and brings several advantages over a parallel group design. (See above).

Use of inactive placebo in pain trials: Ethical guidance does not recommend use of placebo in pain trials when there proven treatments exists, and where head to head comparisons are recommended. However because of a marked placebo effect in pain studies, the use of placebo where there is little or no evidence for treatment is recommended. The magnitude of this placebo effect is also considered in the sample size calculation. In the case of HIV related DSN there is no proven treatment. There are many negative trials for HIV DSN, including amitriptyline.

Pain during washout period: There will be a three weeks washout period between arm A and arm B. The ½ life of amitriptyline is 10-50 hours, with an average of 16 hours. It is eliminated over several days, primarily in the urine as active and inactive metabolites.

Choice of amitriptyline: This is a widely available drug, on the South African EDL. The two previous trials for HIV DSN have shown the drug to be well tolerated. There are no documented or theoretical drug interactions with ARV therapy. Whilst two trials have shown no effect, these were tested on a different population, for all levels pain. Achieving a clinical effect for mild levels of pain may have masked any effect for moderate to severe pain. There

were other limitations to these trials (see above). In addition to this the drug is recommended by the SADoH and the SAHIV Society and the FPD, with little evidence. Other drugs should be used with care because of potential drug interaction with current 1st line ARV therapy (for example sodium valporate and carbamazepine) Studies have shown that 95% of pains in HIV are untreated. There is no standard for the treatment of DSN in HIV at these hospitals. At the palliative care services amitriptyline is offered, but not all patients are routinely assessed and treated for DSN. The evidence is contrary to the routine practice. The trial period is made as short as is efficacious.

Regulatory issues

This trial will be conducted at one academic centre, which includes three HIV clinics and three palliative care clinics. Permission will be sought from one local ethics review committee, the Wits REC. Amitriptyline is registered for the indication of pain relief for chronic pain syndromes. Thus MCC permission is not required in this case. However, the MCC will be notified about intent to conduct this work and will be invited to get further clarification, information and so on. The trial has been registered on the South African Clinical trials register . All three authorities and the study DSMB will receive a report on the completion or the closing of the trial.

Monitoring and DSMB

This trial will be monitored to protect the participants from being exposed risk. An independent data, safety and monitoring board will be constituted to monitor and stop the trial should it become futile; should participants experience frequent or unexpected adverse events; should there be scientific misconduct; protocol violations, for example investigator blinding or randomisation

This will comprise the following members:
A Rice (UK), P Kamerman, Andy Gray, F Venter, K. Zuma (HSRC) and Lynne Coutsalis. They will be provided with an interim progress report, record of adverse events, protocol violations, data report, plus analysis.

The Wits REC shall be provided with a progress report every 6 months. They will receive a detailed record of adverse events serious, protocol violations as they occur. They will also receive and monitor the trial for participant complaints or concerns, and may also recommend early termination of the trial.

Dissemination of information

The information from the study will be communicated to the study participants and shared with the Palliative care centre team. The results will be reported to the national clinical trials register. The results will also be submitted for peer review and publication. This will occur even in the event of a ‘negative’ i.e. no effect trial. The report will be in the CONSORT recommended format. Raw data will be available for public scrutiny on request.

DATA COLLECTION MANAGEMENT AND ANALYSIS

Data will be entered in real time by a data capturer into a Micropsft Access database. 20% of random sample of data will be double entered by a second data enterer and checked for error rate. The double entry will be adjusted according to the error rate. Hard copy of the

consent forms will be kept in a separate locked filing cabinet. Hard copy of the data forms will be kept in another fling cabinet. After closure of the study, forms will be archived and stored for 7 years. Analysis will be in an intention to treat basis. Primary and secondary outcome measures will be calculated and tested for statistical significance by the biostatistician.

DISSEMINATION

Study findings will be written up and sent for publication. The SA national clinical trials register will be notified. The findings will be presented at a relevant conference. If the findings are 'negative' and not accepted for publication in relevant journals , they will be sent to the journal of negative trials. Experience of methodological issues will also be written up for publication.

LOGISTICAL ISSUES

Budget and human resources and infrastructure

CVs, Good Clinical Practice certificates

Timelines

	June 2008	July	August	Sept	Oct	Nov	Dec	Jan	Feb	Mar	April	May	June
Ethical approval	■												
Training of staff		■	■										
recruitment				■									
Enrollment and follow-up					■	■	■	■					
Data Analysis									■	■			
Report Writing											■	■	■
Submission													
Dissemination													

Funding and Conflict of interest

This study is investigator initiated, and funded by the Princess Dian memorial fund on condition that ethical approval is received. Aspen pharmaceuticals, the manufacture of Trepiline® have been approached to manufacture the placebo, which the study will purchase. There is no conflict of interest for study staff or DSMB.

APPENDICES

Patient information sheet for screening
Patient information sheet for study
Informed consent for screening
Informed consent for study
Screening form
Baseline, visit follow up and exit visit forms
SAE, EA, protocol violations, SOPs

REFERENCES

(1) Smyth K, Affandi JS, McArthur JC, Bowtell-Harris C, Mijch AM, Watson K, et al. Prevalence of and risk factors for HIV-associated neuropathy in Melbourne, Australia 1993-2006. HIV Med 2007 Sep;8(6):367-73.
(2) Cornblath DR, Hoke A. Recent advances in HIV neuropathy. Curr Opin Neurol 2006 Oct;19(5):446-50.
(3) Hahn K, Arendt G, Braun JS, von Giesen HJ, Husstedt IW, Maschke M, et al. A placebo-controlled trial of gabapentin for painful HIV-associated sensory neuropathies. J Neurol 2004 Oct;251(10):1260-6.
(4) Simpson DM, Olney R, McArthur JC, Khan A, Godbold J, Ebel-Frommer K. A placebo-controlled trial of lamotrigine for painful HIV-associated neuropathy. Neurology 2000 Jun 13;54(11):2115-9.
(5) Simpson DM, McArthur JC, Olney R, Clifford D, So Y, Ross D, et al. Lamotrigine for HIV-associated painful sensory neuropathies: a placebo-controlled trial. Neurology 2003 May 13;60(9):1508-14.
(6) Shlay JC, Chaloner K, Max MB, Flaws B, Reichelderfer P, Wentworth D, et al. Acupuncture and amitriptyline for pain due to HIV-related peripheral neuropathy: a randomized controlled trial. Terry Beirn Community Programs for Clinical Research on AIDS. JAMA 1998 Nov 11;280(18):1590-5.
(7) Kieburtz K, Simpson D, Yiannoutsos C, Max MB, Hall CD, Ellis RJ, et al. A randomized trial of amitriptyline and mexiletine for painful neuropathy in HIV infection. AIDS Clinical Trial Group 242 Protocol Team. Neurology 1998 Dec;51(6):1682-8.
(8) Canter JA, Haas DW, Kallianpur AR, Ritchie MD, Robbins GK, Shafer RW, et al. The mitochondrial pharmacogenomics of haplogroup T: MTND2*LHON4917G and antiretroviral therapy-associated peripheral neuropathy. Pharmacogenomics J 2008 Feb;8(1):71-7.
(9) Hulgan T, Haas DW, Haines JL, Ritchie MD, Robbins GK, Shafer RW, et al. Mitochondrial haplogroups and peripheral neuropathy during antiretroviral therapy: an adult AIDS clinical trials group study. AIDS 2005 Sep 2;19(13):1341-9.
(10) Woolf CJ, Mannion RJ. Neuropathic pain: aetiology, symptoms, mechanisms, and management. Lancet 1999 Jun 5;353(9168):1959-64.
(11) European Medicines Agency. Guidelines on clinical medicinal products intended for the treatment of neuropathic pain. 2008.
(12) Berger A, Dukes EM, Oster G. Clinical characteristics and economic costs of patients with painful neuropathic disorders. J Pain 2004 Apr;5(3):143-9.
(13) Moog C, Kuntz-Simon G, Caussin-Schwemling C, Obert G. Sodium valproate, an anticonvulsant drug, stimulates human immunodeficiency virus type 1 replication independently of glutathione levels. Journal of General Virology 1996;77(9):1993-9.
(14) Simpson DM, Haidich AB, Schifitto G, Yiannoutsos CT, Geraci AP, McArthur JC, et al. Severity of HIV-associated neuropathy is associated with plasma HIV-1 RNA levels. AIDS 2002 Feb 15;16(3):407-12.

(15) Murphy RA, Sunpath H, Kuritzkes DR, Venter F, Gandhi RT. Antiretroviral therapy-associated toxicities in the resource-poor world: the challenge of a limited formulary. J Infect Dis 2007 Dec 1;196 Suppl 3:S449-S456.
(16) Kieburtz K, Simpson D, Yiannoutsos C, Max MB, Hall CD, Ellis RJ, et al. A randomized trial of amitriptyline and mexiletine for painful neuropathy in HIV infection. AIDS Clinical Trial Group 242 Protocol Team. Neurology 1998 Dec;51(6):1682-8.
(17) Cherry CL, Wesselingh SL, Lal L, McArthur JC. Evaluation of a clinical screening tool for HIV-associated sensory neuropathies. Neurology 2005 Dec 13;65(11):1778-81.
(18) Cherry CL, Skolasky RL, Lal L, Creighton J, Hauer P, Raman SP, et al. Antiretroviral use and other risks for HIV-associated neuropathies in an international cohort. Neurology 2006;66(6):867-73.
(19) Wester CW, Okezie OA, Thomas AM, Bussmann H, Moyo S, Muzenda T, et al. Higher-than-expected rates of lactic acidosis among highly active antiretroviral therapy-treated women in Botswana: preliminary results from a large randomized clinical trial. J Acquir Immune Defic Syndr 2007 Nov 1;46(3):318-22.
