# Supplementary material for: Randomized, Double-Blind, Crossover Trial of Amitriptyline for Analgesia in Painful HIV-Associated Sensory Neuropathy
Source: PLoS One. 2015 May 14;10(5):e0126297. doi: 10.1371/journal.pone.0126297 (PMC4431817; doi:10.1371/journal.pone.0126297)
Supplement: S1 Table — (PDF) [file pone.0126297.s008.pdf]

S1 Table. ANOVA summary (intention-to-treat cohort, n=124)

	df	F-value	p-value
**ARV-USER**			
**Between group**			
Order[1]	1, 60	0.04	0.85
**Within group**			
Period[2]	1, 306	92.8	< 0.001*
Time[3]	2, 306	37.0	< 0.001*
Treatment[4]	1, 306	2.4	0.13
**ARV-NAIVE**			
**Between group**			
Order[1]	1, 60	0.02	0.89
**Within group**			
Period[2]	1, 306	115.3	< 0.001*
Time[3]	2, 306	47.0	< 0.001*
Treatment[4]	1, 306	0.59	0.44
**ALL PARTICIPANTS**			
**Between group**			
Order[1]	1, 121	0.03	0.86
ARV[5]	1, 121	3.61	0.06
**Within group**			
Period[2]	1, 616	208.4	< 0.001*
Time[3]	2, 616	84.0	< 0.001*
Treatment[4]	1, 616	2.74	0.1

[1] Order of treatment; [2] Period 1 vs. period 2; [3] Weeks; [4] Amitriptyline vs. placebo; [5] ARV-user vs. ARV-naïve; [*] Statistically significance
