# Supplementary material for: Randomized, Double-Blind, Crossover Trial of Amitriptyline for Analgesia in Painful HIV-Associated Sensory Neuropathy
Source: PLoS One. 2015 May 14;10(5):e0126297. doi: 10.1371/journal.pone.0126297 (PMC4431817; doi:10.1371/journal.pone.0126297)
Supplement: S2 Table — (PDF) [file pone.0126297.s009.pdf]

S2 Table. ANOVA summary for period 1 comparison of amitriptyline and placebo-treated participants (per protocol cohort, n=122)

	df	F-value	p-value
**Between group**			
Treatment[1]	1, 120	0.63	0.43
**Within group**			
Time[2]	2, 242	53.9	< 0.001*

[1] Amitriptyline vs. placebo; [2] Weeks; [*] Statistically significant
