# Supplementary material for: Randomized, Double-Blind, Crossover Trial of Amitriptyline for Analgesia in Painful HIV-Associated Sensory Neuropathy
Source: PLoS One. 2015 May 14;10(5):e0126297. doi: 10.1371/journal.pone.0126297 (PMC4431817; doi:10.1371/journal.pone.0126297)
Supplement: S3 Table — (PDF) [file pone.0126297.s010.pdf]

S3 Table. Adverse events during amitriptyline and placebo treatment (n=122)

	Placebo [n (%)]	Amitriptyline [n (%)]	p-value[*]
**Dry mouth**	1 (0.8)	9 (7.4)	<0.01
**Drowsiness**	3 (2.5)	7 (5.7)	0.34
**Chest Pain**	1 (0.8)	0 (0)	1.00

[*] McNemar's test
