# Supplementary material for: Randomized, Double-Blind, Crossover Trial of Amitriptyline for Analgesia in Painful HIV-Associated Sensory Neuropathy
Source: PLoS One. 2015 May 14;10(5):e0126297. doi: 10.1371/journal.pone.0126297 (PMC4431817; doi:10.1371/journal.pone.0126297)
Supplement: S4 Table — (PDF) [file pone.0126297.s011.pdf]

Table S4. Rescue medications (per protocol cohort, n = 122)

	Placebo [n (%)]	Amitriptyline [n (%)]	p-value[1]
**Took rescue medication for pain in the last week of each intervention period**	13 (10.7)	13 (10.7)	1.0
**Number pain medications listed on participants' drug inventories**			
0	19 (15.6)	21 (17.2)	
1	83 (68.0)	81 (66.4)	
2	20 (16.4)	20 (16.4)	
**Analgesics and anti-inflammatories listed on participants' drug inventories**			
Paracetamol	78 (63.9)	78 (63.9)	1.0
NSAID[2] or aspirin	32 (26.2)	25 (20.5)	0.35
Paracetamol with codeine	7 (5.7)	10 (8.2)	0.18
Paracetamol/aspirin/caffeine	6 (4.9)	8 (6.6)	0.32

[1] p-values were obtained using McNemar's test for paired nominal data, which tests the null hypothesis of equal probabilities of rescue medication in the placebo and amitriptyline interventions respectively.

[2] Non-steroidal anti-inflammatory drugs
